# ATM phosphorylates PP2A subunit A resulting in nuclear export and spatiotemporal regulation of the DNA damage response

**DOI:** 10.1007/s00018-022-04550-5

**Published:** 2022-11-24

**Authors:** Amrita Sule, Sarah E. Golding, Syed F. Ahmad, James Watson, Mostafa H. Ahmed, Glen E. Kellogg, Tytus Bernas, Sean Koebley, Jason C. Reed, Lawrence F. Povirk, Kristoffer Valerie

**Affiliations:** 1 Department of Radiation Oncology, Virginia Commonwealth University, Richmond, VA 23298-0058 USA; 2 Department of Biochemistry and Molecular Biology, Virginia Commonwealth University, Richmond, VA 23298 USA; 3 Department of Medicinal Chemistry, Virginia Commonwealth University, Richmond, VA 23298 USA; 4 Department of Anatomy, Virginia Commonwealth University, Richmond, VA 23298 USA; 5 Department of Physics, Virginia Commonwealth University, Richmond, VA 23298 USA; 6 Department of Pharmacology and Toxicology, Virginia Commonwealth University, Richmond, VA 23298 USA; 7 Massey Cancer Center, Virginia Commonwealth University, Richmond, VA 23298 USA

**Keywords:** CRM1, DNA repair, Mitotic catastrophe, Nuclear export, HS-AFM, Radiation

## Abstract

Ataxia telangiectasia mutated (ATM) is a serine–threonine protein kinase and important regulator of the DNA damage response (DDR). One critical ATM target is the structural subunit A (PR65–S401) of protein phosphatase 2A (PP2A), known to regulate diverse cellular processes such as mitosis and cell growth as well as dephosphorylating many proteins during the recovery from the DDR. We generated mouse embryonic fibroblasts expressing PR65-WT, -S401A (cannot be phosphorylated), and -S401D (phospho-mimetic) transgenes. Significantly, S401 mutants exhibited extensive chromosomal aberrations, impaired DNA double-strand break (DSB) repair and underwent increased mitotic catastrophe after radiation. Both S401A and the S401D cells showed impaired DSB repair (nonhomologous end joining and homologous recombination repair) and exhibited delayed DNA damage recovery, which was reflected in reduced radiation survival. Furthermore, S401D cells displayed increased ERK and AKT signaling resulting in enhanced growth rate further underscoring the multiple roles ATM–PP2A signaling plays in regulating prosurvival responses. Time-lapse video and cellular localization experiments showed that PR65 was exported to the cytoplasm after radiation by CRM1, a nuclear export protein, in line with the very rapid pleiotropic effects observed. A putative nuclear export sequence (NES) close to S401 was identified and when mutated resulted in aberrant PR65 shuttling. Our study demonstrates that the phosphorylation of a single, critical PR65 amino acid (S401) by ATM fundamentally controls the DDR, and balances DSB repair quality, cell survival and growth by spatiotemporal PR65 nuclear–cytoplasmic shuttling mediated by the nuclear export receptor CRM1.

**Supplementary Information:**

The online version contains supplementary material available at 10.1007/s00018-022-04550-5.

## Introduction

Protein phosphatase 2A (PP2A) is a major serine/threonine (S/T) phosphatase that regulates many cellular processes including DNA replication, cell cycle progression, cell differentiation, and the DNA damage response (DDR) [1–3]. The mammalian PP2A holoenzyme is a hetero-trimer (ABC) comprises a scaffolding subunit (A or PR65), a regulatory subunit (B), and a catalytic subunit (C), or a heterodimer (AC; PP2A Core enzyme) [4]. Both the A and C subunits have two isoforms that are evolutionary conserved [5, 6]. The two PR65 isoforms, Aα (PPP2R1A) and Aβ (PPP2R1B) are 86% identical. Each PR65 isoform is shaped into a horseshoe composed of 15 nonidentical internal repeats of a 39 amino acid sequence, termed HEAT (Huntingtin, Elongation factor 3, Protein phosphatase 2A-subunit A, and the yeast kinase TOR1) motif [7–9]. Almost 90% of the PP2A holoenzymes have the Aα isoform whereas Aβ is primarily associated with development and the CNS [10]. There are at least 17 subtypes of the B subunit, classified into four distinct families, B/PR55, B’/PR61, B’’/PR72 and PTP/PR53 [4, 6, 11, 12]. Specific regulatory B subunits control the diversity and target specificity of the various PP2A holoenzymes. The association of the AC heterodimer with a specific B subunit imparts complete activity, subcellular localization, and substrate specificity to the heterotrimeric holoenzyme.

Ataxia telangiectasia mutated (ATM), an S/T kinase, is defective in the human autosomal recessive disorder ataxia telangiectasia (A–T) and is one of the major regulators of the cellular response to DNA damage [13]. ATM is activated by DNA double-strand breaks (DSBs), induced endogenously by growth factor signaling, and exogenously by DNA damaging agents like ionizing radiation (IR) and radiomimetic drugs. In the event of DNA damage, ATM and other PIKKs such as A–T and RAD3-related (ATR), and DNA-PKcs get activated and together direct a coordinated response referred to as the DDR by acting at several levels, including cell cycle checkpoints, DNA repair, and apoptosis [13–18]. In a sense, ATM acts as a cellular rheostat balancing and coordinating the DDR with cell growth to maintain homeostasis [19]. DDR proteins, phosphorylated in response to DNA damage are subsequently dephosphorylated by phosphatases to reverse the DDR and reset the cell to normalcy [14, 20]. We have previously reported that the inhibition of ATM kinase blocks prosurvival signaling in human tumor cell lines by reducing AKT (S473) phosphorylation [21]. This study revealed that ATM regulates AKT phosphorylation via an okadaic acid (OA) sensitive phosphatase. OA inhibits the S/T protein phosphatases protein phosphatase 1 (PP1) and PP2A, exhibiting greater affinity and inhibition towards the latter [22–24].

Protein phosphatase 2A regulates key DDR proteins ATM, ATR, CHK1, CHK2, and p53 [25–28], controls the G2/M checkpoint [29], and facilitates DSB repair via the dephosphorylation of γ-H2AX in response to IR [30]. More specifically, PP2A binds to ATM and regulates ATM (S1981) autophosphorylation and activation in an indirect manner [25]. Furthermore, it was reported that PP2A is regulated by ATM-mediated phosphorylation of PR65 at S401 resulting in the retention of histone deacetylase HDAC4 in the cytoplasm [31]. Consequently, without functional ATM, HDAC4 is unable to shuttle between the cytoplasm and nucleus where it permanently remains resulting in neurodegeneration which is a characteristic hallmark of A–T. Even though this study did not address the impact of PR65 S401 (de)phosphorylation during the DDR, a major conclusion from the report was that PP2A is negatively regulated by ATM, which fits well with how we perceive ATM to regulate PP2A and AKT phosphorylation [21].

In the present study we generated PR65 depleted mouse embryonic fibroblasts (MEFs) expressing PR65 site-specific S401 mutants; a S401A mutant which cannot be phosphorylated as well as a phospho-mimetic S401D mutant. We found that these mutant cells were impaired in the DDR, displayed increased radio- and chemo-sensitivity, and had fundamentally altered DSB repair. Both S401 mutants were deficient in HRR with S401D also being severely compromised in nonhomologous end joining (NHEJ) whereas S401A appeared to possess aberrant, greater than normal end joining activity. Upon assessing further the phenotype of these cells, we uncovered a completely rewired DSB repair machinery due to PP2A malfunctioning. Here we report that the spatial and temporal regulation of PR65 phosphorylation at S401 by ATM resulting in nuclear–cytoplasmic shuttling is critical for regulating DSB repair quality and the proliferative recovery from the DDR.

## Materials and methods

### Generation of PR65 conditional knockout mouse embryonic fibroblasts

All animal breeding and experiments were approved by the Virginia Commonwealth University IACUC. Fvb.129S-*PPP2R1A*^*tm1.1Wltr/j*^ mice [32] were obtained from Jackson laboratories as stock number 017441. These CKO mice possess *lox*P sites flanking exons 5–6 of the PR65 gene (*PPPP2R1A*). Heterozygous 129S-*PPP2R1A*^*tm1.1Wltr/j*^ (CKO/WT) mice were bred to obtain 129S-*PPP2R1A*^*tm1.1Wltr/tm1.1Wltr*^ (CKO/CKO) mice. Embryos were harvested at 13.5 dpc from a pregnant CKO/CKO female mouse and PR65–CKO MEFs generated by the VCU Massey Cancer Center Transgenic/Knockout Mouse Core using standard methods [33].

### Cell culture and treatments

HEK293, HEK293T, U1242, U87 glioma cells, and MEFs were cultured in DMEM (GIBCO) medium supplemented with 10% FBS and 1% Pen–Strep (Life Technologies). Cell treatments were as described in the figure legends. Serum starvation was for 16 h in medium without FBS. Irradiations were done using an MDS Nordion Gammacell-40 research irradiator with 137-Cs source delivering a dose rate of 1.05 Gy/min. Cell growth was determined by CellTiter-Glo^®^ Luminescent Cell Viability Assay (Promega, Cat #G7570). Cells were serially diluted and seeded in a 96-well plate. At days 2, 5 and 7 after seeding, CellTiter-Glo^®^ reagent was added to the medium at the recommended final concentration. Plates were incubated for 10 min at room temperature and luminescence determined using an EnVision Multilabel reader (PerkinElmer) and the readings taken as the measure of cell growth. Clonogenic survival assays were carried out as described [34, 35].

MEFs were arrested in mitosis by treating cells with 50 ng/ml of nocodazole for 16 h. After nocodazole washout, cells were collected for western blotting at 1, 2 and 4 h with p-PLK1 (T210) antibody. MEFs were treated with 1 mM of hydroxyurea (HU) for 2 h followed by washout and cells collected at 0, 1, and 6 h for western blotting with antibodies specific for pRPA (S4/S8) and γ-H2AX.

### Plasmids

Previously described pMIG-Aalpha WT retroviral vector expressing PR65 with a Flag-tag at the NH_2_ terminal was used as a starting point for generating all PR65 plasmids [36]. Mutations at the S401 codon and other positions were generated using QuikChange site-directed mutagenesis (Stratagene). The PR65 mutations were verified by DNA sequencing. A complete list of plasmids is shown in the Supplementary Information (SI).

### Antibodies and other reagents

Antibodies for western blotting and/or immunocytochemistry were used as described [21, 37, 38] and are listed in the SI.

Additional Materials and Methods can be found in the SI.

### Statistics

Unpaired two-tailed *t* tests or one-way ANOVA were performed on triplicate or more data sets using GraphPad Prism 3.0 (Graphpad Software, inc.). *p* values are indicated as *< 0.05; **< 0.01; and ***< 0.001.

## Results

### PP2A functions downstream of ATM to regulate AKT

In a previous study from our laboratory, we demonstrated that ATM indirectly regulates the phosphorylation of AKT at S473 via an okadaic acid (OA) sensitive phosphatase [21]. It is known that several oncogenic DNA viruses such as SV40 and Polyoma interfere with PP2A activity in infected cells through the expression of small t-antigen and PyMT that block PP2A activity by replacing the B subunit [39–41] (Supplementary Fig. S1A). After examining the effect of an ATM kinase inhibitor (ATMi) in a panel of tumor cell lines including human U87 and U1242 glioma cells as well as HEK293 and HEK293T carcinoma cells, inhibition of AKT phosphorylation was observed across the board relative to untreated cells with the exception of HEK293T cells (Fig. S1B). HEK293T expresses SV40 t/T antigens, which inversely correlates with the lack of pAKT inhibition by ATMi, suggesting similar or overlapping mechanism of action. To build on this finding, we infected U87 and U1242 cells with retroviruses expressing PyMT or not (Fig. S1C, D). ATMi did not appear to reduce the levels of pAKT in glioma cells overexpressing PyMT and was unaffected by ionizing radiation (IR) [21, 42]. The presence of the PyMT gene in these cell populations was verified by PCR (Fig. S1E). We confirmed the ability of OA to increase the levels of phosphorylated AKT [21], this time in a nuclear extract with nM concentrations suggesting that PP2A is acting on AKT in the nucleus (Fig. S1F), perhaps as a complex in direct physical association with AKT [43]. Altogether, these results suggest that ATM regulates the phosphorylation of AKT indirectly via the inhibition of PP2A activity.

### Generation of PR65 KO MEFs complemented with S401 mutant alleles

**Fig. 1:**
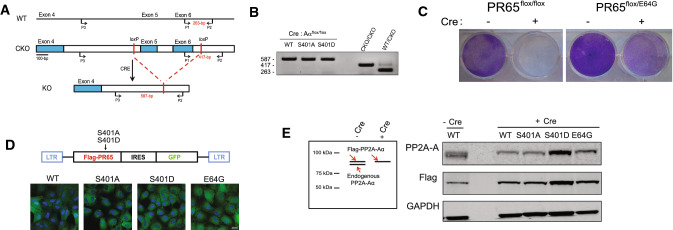
Generation of PR65 KO MEFs complemented with S401 mutants. **A** Targeting strategy for generating PR65 conditional knockout (CKO) MEFs. Expression of Cre recombinase in these PR65-CKO MEFs would generate PR65 KO MEFs by the deletion of exon 5 and 6. PR65-CKO MEFs were infected with retroviral vector pMIG-Aalpha, expressing Flag-tagged PR65 WT, S401A or S401D mutant in addition to GFP. This was followed by infection of cells with Ad-CMV-Cre recombinase to knock out the endogenous PR65 alleles. **B** Genomic DNA was isolated from PR65 KO MEFs (WT, S401A and S401D) and PCR amplified. PCR screening with primers P1, P2, and P3 shown in **A** generated a 263-bp product for the WT allele and a 417-bp product for the CKO allele. In cells where Cre was expressed, primer pair P2–P3 generated a 587-bp product for the KO PR65 allele. **C** PR65 is essential for the survival of MEFs. PR65 CKO MEFs were infected with an adenovirus expressing Cre recombinase. Cells were stained with crystal violet after one week to assess cell viability. PR65–CKO MEFs infected with Ad–CMV–Cre did not survive whereas MEFs complemented with PR65^flox/E64G^ did. PR65^flox/E64G^ MEFs express a known human tumor-associated PR65 mutant [32], constructed the same as S401A and S401D MEFs, and here used as control cells expressing mutant PR65. **D** Plasmid map of pMIG-Aalpha WT with mutations marked (top) and expression of GFP via an IRES in WT, S401A, S401D, and E64G MEFs with endogenous *PR65* alleles KO. Scale bar is 10 μm. **E** Western blot verifying the Flag-PR65 expression of site-specific mutants and floxing of endogenous PR65 in MEFs. Left panel: explanation of the protein bands seen by western blotting. Right panel: whole cell extracts of GFP+ sorted PR65 WT, S401A and S401D cells infected with Ad-CMV-Cre or not were separated on an SDS-PAGE gel and analyzed by western blotting using anti-PR65 antibody, anti-Flag and anti-GAPDH (loading control) antibodies

Ruediger et al. demonstrated that PR65 is an essential gene, since knocking out PR65 resulted in embryonic lethality [32]. We generated homozygous PR65 conditional knockout (CKO) mouse embryonic fibroblasts (MEFs) after breeding heterozygous 129S-*PPP2R1A*^*tm1.1Wltr/j*^ mice to obtain 129S-*PPP2R1A*^*tm1.1Wltr/tm1.1Wltr*^ (CKO/CKO) mice. Floxing out PR65 exon 5 and 6 generates PR65 KO MEFs (Fig. 1A). MEFs generated from 129S-*PPP2R1A*^*tm1.1Wltr/tm1.1Wltr*^ mouse embryos were immortalized by passaging once per week [33], and infecting the cells with a lentivirus expressing human TERT. We generated two site-specific mutations at the S401 codon of PR65 using the retroviral vector pMIG-Aalpha WT as template [36]; a S401A mutant that cannot be phosphorylated and a S401D phospho-mimetic mutant. The MEFs were then separately infected with the retroviruses expressing Flag-tagged PR65 WT, S401A or a S401D mutant in addition to GFP (via an IRES). The endogenous PR65 alleles were then floxed out by infecting the cells with an adenovirus expressing Cre recombinase (Ad–Cre). Complete floxing was confirmed by PCR (Fig. 1B). MEFs that were not complemented with virus expressing PR65 did not survive after infection with Ad–Cre (Fig. 1C), in line with that PR65 is essential in the mouse [32]. Pooled MEFs from each infection were shown to be close to 100% GFP+ (Fig. 1D). Western blotting with anti-PR65 and -Flag antibodies showed that at the protein level all endogenous PR65 (Fig. 1E, *left panel; lower band*), was replaced with the slightly larger Flag-PR65 protein expressed from the retrovirus. Altogether, we generated a panel of isogenic WT, S401A, and S401D cell populations expressing PR65 at approximately the same levels as endogenous PR65 (compare band intensity of PR65 doublet (Fig. 1E, right panel; lane 1).

### ATM kinase phosphorylates PR65 in vitro

The PR65 S401 residue is part of an –S/Q-ATM kinase signature motif highly conserved in PR65’s among mammals including human and mouse and is a *bona fide* substrate for ATM phosphorylation [31]. To obtain an abundant source of co-expressed ATM and PR65 for further analysis and experimentation, we first attempted to isolate stably transfected HEK293 cells co-expressing mRuby2–PR65 and YFP–ATM after selection with G418 (expressed from YFP–ATM) and isolating cells expressing both YFP and Ruby. However, we were only able to recover YFP–ATM/S401A cells (Fig. S2A), suggesting that the overexpression of ATM with either S401D or WT was not tolerated.

To determine whether ATM phosphorylates PR65 in vitro and to confirm previously documented results [31], YFP–ATM was immunoprecipitated from HEK293 cells expressing YFP–ATM only (Fig. S2B) and utilized for in vitro kinase assays with [γ-^32^P] ATP and GST-PR65_FL_ as substrate. The result shows that ATM kinase phosphorylates PR65 in vitro and that phosphorylation is significantly reduced in the presence of an ATMi (Fig. S2C, compare lanes 5 and 6). Similarly, GST-p53_1–100_, a known substrate of ATM kinase at p53 S15 [44], was also phosphorylated and inhibited in the presence of the ATMi. This result confirms that ATM kinase is able to phosphorylate PR65 in vitro in line with a previous report [31].

### A phospho-mimetic (S401D) PR65 mutant prevents the association with ATM, PP2A catalytic subunit and AKT

**Fig. 2:**
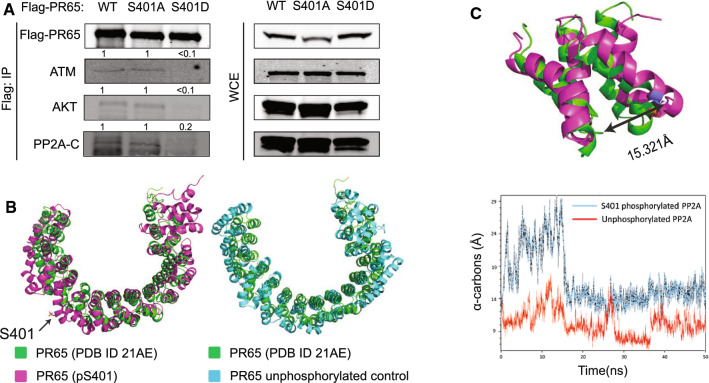
Phospho-mimetic S401D prevents the association of PR65 with ATM, AKT, and PP2A catalytic subunit. **A** Whole cell lysates of HEK293 cells stably expressing Flag-PR65 (WT, S401A and S401D) were immunoprecipitated with anti-Flag beads. Immunoprecipitates (left panel) and whole cell extracts (right panel) were separated on an SDS-PAGE gel and analyzed by western blotting using anti-Flag, anti-ATM, anti-AKT, and anti-PP2A-C antibodies. Fold protein levels relative to WT is shown above each panel. **B** MD simulations suggest that a conformational change occurs when S401 is phosphorylated. (Left) Top view of the in silico S401 phosphorylated PR65, after 50 ns, aligned to the original experimental structure (PDB ID 2IAE) with a root-mean square deviation (RMSD) of 8.05 Å. PR65 (pS401) is shown in magenta, with pS401 represented as stick figures. PR65 from protein database (PDB ID 21AE) is shown in green. (Right) Top view of the unphosphorylated control structure of PR65 subunit, after 50 ns, aligned to the original experimental structure taken from PDB ID 2IAE (RMSD = 6.51 Å). Unphosphorylated control PR65 is shown in cyan, and the original experimental structure shown in green. Molecular modeling studies also indicate that phosphorylation at S401 could cause a conformational change which could affect its association with a binding partner. **C** Two 50 ns molecular dynamics simulations were carried out using the S401 phosphorylated PR65 chain as well as the unphosphorylated structure. Comparing only the amino acid residues surrounding S401 (residues 375–454), the RMSD of the phosphorylated subunit and the unphosphorylated experimental structure was found to be 4.448 Å while the RMSD of the control and the unphosphorylated experimental structure was 2.55 Å. At the end of the simulation, the distance between α-carbons for residues S401 and A431 for the unphosphorylated original structure (PDB ID 2IAE), the S401 phosphorylated structure, and control structure were 10.873 Å (green), 15.321 Å (magenta) and 9.498 Å (cyan), respectively, with the first two structures shown (top panel). Initially, there was a readjustment phase in the phosphorylated MD simulation in the region surrounding the phosphorylated S401 residue after which it stabilized at 18 ns (bottom panel). Altogether, this result suggests that phosphorylation of PR65–S401 could affect the interaction with its binding partners and result in the dissociation of the holoenzyme

Goodarzi et al. showed that PP2A exerts its control over ATM by interacting via the PR65 subunit in a manner lost in irradiated cells [25]. To determine whether the S401D amino acid replacement affected the interaction with ATM, we immunoprecipitated over-expressed Flag-PR65 WT, -S401A, and -S401D, respectively, from transduced HEK293 cells. Flag-PR65 IP’s were then analyzed for the presence of endogenous ATM, the catalytic subunit of PP2A (PP2A-C), and AKT. We found an association of ATM, PP2A-C, and AKT with the WT and the nonphosphorylatable S401A but not with S401D (Fig. 2A). The interaction of ATM with PR65-WT and -S401A, but not -S401D, suggests that the PP2A Core enzyme (AC) is in a complex with ATM, in agreement with previous work [25]. That the S401D phospho-mimetic disrupts this complex is new information and fits well with the dissociation of ATM and PP2A complex after irradiation and the phosphorylation of PR65 S401 by ATM [25, 31]. That AKT is found complexed with PR65 is no surprise [43], but that S401D disrupts this interaction is intriguing, even though we cannot tell from this result whether ATM and AKT are in the same or separate PR65 complexes.

To support a critical role for S401 phosphorylation by ATM in the disruption of such physical interaction we performed molecular modeling studies based on the known PR65 crystal structure (PDB ID 2IAE). Two 50 ns molecular dynamics runs were carried out with phosphorylated S401 PR65 as well as the unphosphorylated structure (Fig. 2B, C). Our result suggests that phosphorylation at S401 induces a local movement of the α-helices on either side of the helix (residues 375–454) encompassing S401 ultimately causing a conformational change of the entire PR65 protein, particularly at its N-terminal portion. This conformational change results in the distortion of the horseshoe shape of the subunit by 4.448 Å as compared to the unphosphorylated structure (15.321–10.873). Altogether, the modeling suggests that phosphorylation at S401 could cause the dissociation of the physical interaction with its binding partners ATM and AKT.

### Growth and radiation signaling are altered in S401 mutant cells

We then examined whether AKT signaling and growth was affected in response to insulin stimulation and radiation [21]. WT, S401D, and S401A MEFs were serum-starved prior to stimulation with insulin followed by western blotting for pAKT (S473) (Fig. S3). We found that S401D cells responded more robustly to insulin when pAKT levels were determined over the course of 60 min (Fig. S3A), which correlated with an elevated growth rate of S401D relative to WT and S401A cells (Fig. S3B). Similar to AKT signaling, ERK signaling is associated with prosurvival and increased cell growth after low radiation doses, which in part is regulated by ATM [45, 46]. When we examined ERK signaling in unstimulated cells we observed elevated pERK1/2 levels in S401A and more so in S401D cells compared with WT, suggesting that AKT and ERK signaling are positively affected when S401 is mutated, with S401D cells being affected the most (Fig. S3C). Furthermore, after a single radiation dose of 2 Gy, S401 mutant cells showed dampened responses when analyzed for both γ-H2AX and pAKT by western blotting (Fig. S3D). It is well-established that ATM phosphorylates H2AX at S139 (γ-H2AX) in response to radiation at the sites of DSBs [47]. Furthermore, PP2A modulates DSB repair via the dephosphorylation of γ-H2AX to reverse chromatin accessibility [30]. On examining the γ-H2AX levels in more detail, we found that in WT cells the levels of γ-H2AX peaked at 15–30 min post irradiation and then declined. However, H2AX phosphorylation was reduced in both S401A and S401D cells with the latter trending even higher than WT and S401A at 60 min (Fig. S3D). Similarly, pAKT levels increased after IR in WT and less so in S401A and S401D cells. These results suggest that after low dose radiation, S401 mutant cells have dampened pAKT and γ-H2AX levels likely as a result of malfunctioning DDR. In addition, S401D seems to overcome this initial blockade with a delayed response and increased γ-H2AX levels at later times. Altogether, we conclude that S401 mutant cells have aberrant responses to insulin and radiation yet distinct from each other, resulting in altered DDR signaling and proliferative activity compared with WT cells. We recognize that these experiments need further in-depth investigation and analyses.

### PR65 mutants have impaired G2/M checkpoint resulting in aberrant mitosis and elevated levels of mitotic catastrophe

**Fig. 3:**
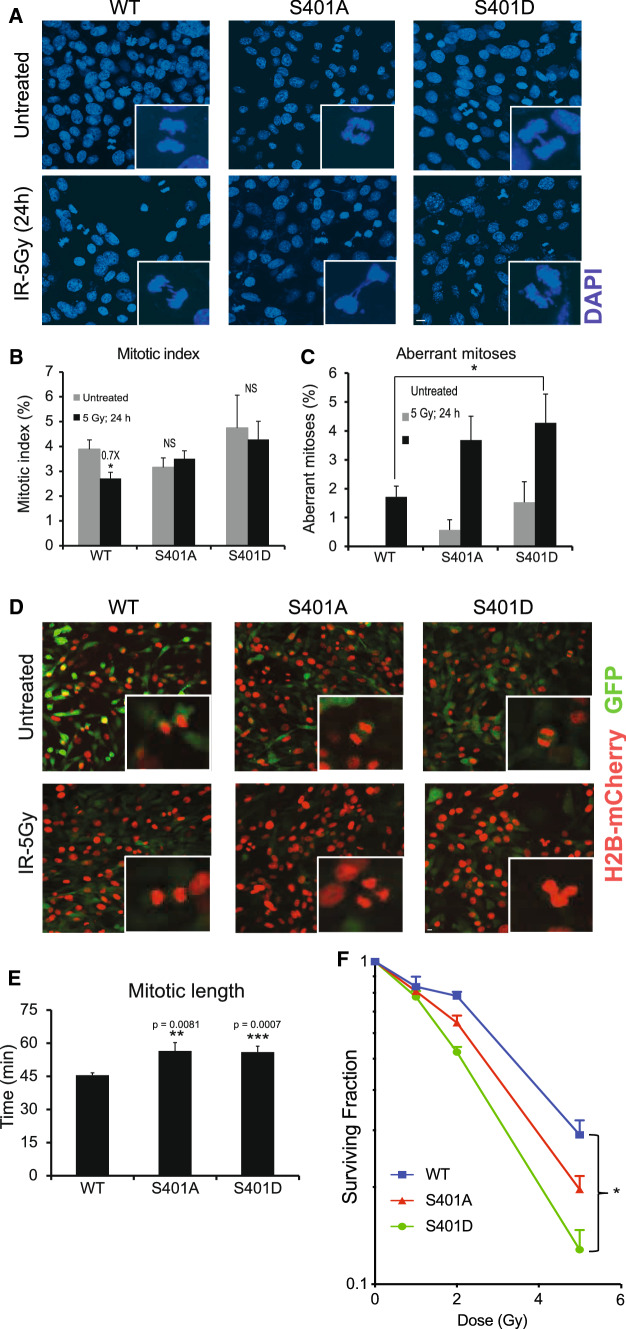
PR65 mutants accumulate mitotic aberrations leading to mitotic catastrophe and radiosensitization. **A**–**C** S401 mutant cells have defective G2/M checkpoint. MEFs were exposed to 5 Gy of ionizing radiation, fixed after 24 h and stained with DAPI to label nuclei. Mitotic cells and total number of cells were counted. The mitotic index was calculated by dividing the number of cells undergoing mitosis in a population to the number of cells not undergoing mitosis. Five separate fields were assessed for each group Scale bar is 10 μm. **B** Mitotic indices were quantified from **A**. Statistical analysis was carried out using unpaired, two-tailed *t* tests. Error bars; mean ± SEM. *p* values expressed as *(*p* < 0.05) were considered significant; NS, not significant (*p* > 0.05). WT: untreated vs IR; *p* = 0.0251. **C** Aberrant mitoses were quantified from **A**. Five separate fields were assessed for each group (*n* = 5)*.* Error bars; mean ± SEM. Statistical analysis was carried out using unpaired, two-tailed *t* tests. *p* values expressed as *(*p* < 0.05) were considered significant; NS, not significant (*p* > 0.05). IR: WT vs S401D; *p* = 0.0228 **D** MEFs were infected with a virus expressing H2B–mCherry to monitor chromatin structure in mitosis. MEFs were exposed to 5 Gy and subjected to live-cell video imaging for 8 h on the Zeiss Cell Observer Spinning Disc confocal microscope. Representative still images (**D** and quantified mitotic length **E** are shown. Scale bar is 10 μm. **F** S401 mutant cells are more radiosensitive than wild-type MEFs. MEFs were exposed to 1, 2 or 5 Gy and radiosurvival determined at 10 days. Surviving fractions were determined by crystal violet staining and colony counting. Data points, surviving cells plotted as fraction of control (- irradiation). The results are presented as mean ± SEM, (*n* = 4). Statistical analysis was carried out with longitudinal ANOVA on the survival fractions. *p* values expressed as *(*p* < 0.05) were considered significant. WT vs. D, *p* = 0.0302

PP2A plays distinct roles at different stages of mitosis while associated with different B subunits [48, 49]. PP2A regulates mitotic entry and exit, as well as playing a role in the protection of centromeres by inhibiting PLK1 and Aurora A kinases [50–54]. Taking into consideration the importance of PP2A in mitosis and elsewhere in the cell cycle, we examined whether S401 mutant cells show any chromosomal aberration or perturbations in mitosis and cell cycle checkpoints. We found elevated basal and radiation-induced levels of chromosomal aberrations in S401 mutants (Fig. 3A). The mitotic index (cells undergoing mitosis/total number of cells) was reduced in WT cells after radiation but not in S401 mutant cells, suggesting that the radiation-induced G2/M check point is intact in WT cells, as expected, but not functioning properly in the mutants (Fig. 3B). Furthermore, S401 mutants had significantly increased numbers of basal and radiation-induced (2- to 3-fold) aberrant mitoses (Fig. 3C). To support this finding, live cell imaging of MEFs expressing a histone H2B-mCherry reporter revealed that the S401 mutant cells exhibited abnormal mitoses including the formation of micronuclei and elevated levels of mitotic catastrophe, and the formation of tetraploid cells whose frequency increased after exposure to radiation (Fig. 3D). Again, S401D cells showed more aberrations than S401A. Furthermore, in undamaged cells, the length of mitosis in S401 mutant cells was significantly longer than in WT cells (Fig. 3E). To examine mitosis in more detail, we then focused on exit from mitosis. PLK1 is known to be critical during G2/M entry as well as for successful chromosome separation and exit from mitosis [55]. Normally, pPLK1 (T210) is elevated in mitosis and upon exit is dephosphorylated by PP2A [56]. A nocodazole blockade synchronized the cell panel in mitosis and following washout (Fig. S4A), we collected cells for western blotting with anti-pPLK1 (T210) antibody (Fig. S4B). Mitotic S401 mutant cells showed elevated pPLK1 levels compared with WT at 1 h and exited mitosis faster than WT cells with S401D showing the most pronounced effect (Fig. S4C). Together, both mitotic entry and exit are abnormal in S401 mutant cells as reflected in significantly longer mitosis and clear signs of struggle to get through mitosis.

To get better insight into what occurs during S-phase we then examined the recovery of collapsed replication forks in the cell panel by determining pRPA32 (S4/8) and γ-H2AX levels after the release from a hydroxyurea (HU) blockade (Fig. S4D, top panel). A previous study showed that PP2A is critical for the dephosphorylation of pRPA32, cell cycle reentry and efficient DSB repair presumably by HRR [57]. We found that S401 mutant cells had reduced γ-H2AX levels after HU release, which peaked at ~ 1 h post washout (Fig. S4D, bottom left panel). Perhaps more importantly, S401D cells showed a delay in the removal of pRPA32 (Fig. S4D, bottom right panel) indicative of an alteration in PP2A activity during fork recovery. In addition, a colony-forming radiosurvival experiment demonstrated that S401D cells were also significantly more radiosensitive than WT cells, with S401A trending more radiosensitive (Fig. 3F). The same pattern of sensitivity was seen when the cell panel was exposed to camptothecin (CPT), a topoisomerase I inhibitor, known to produce DSBs preferentially in S and G2 (Fig. S5).

Altogether, increased chromosomal aberrations seen in untreated S401 mutant cells (and more so after radiation) are suggestive of impaired G2/M checkpoint and aberrant mitosis (entry, duration, and exit), resulting in elevated levels of mitotic catastrophe and increased sensitivity to DNA damage. In addition, replication fork recovery and DSB repair were impaired, especially in the S401D cells. It was however unexpected to see that the impact on survival was relatively minor considering the major negative effects on the G2/M checkpoint and increased mitotic struggle seen in S401 mutant cells.

### S401 mutants have aberrant DSB repair

**Fig. 4:**
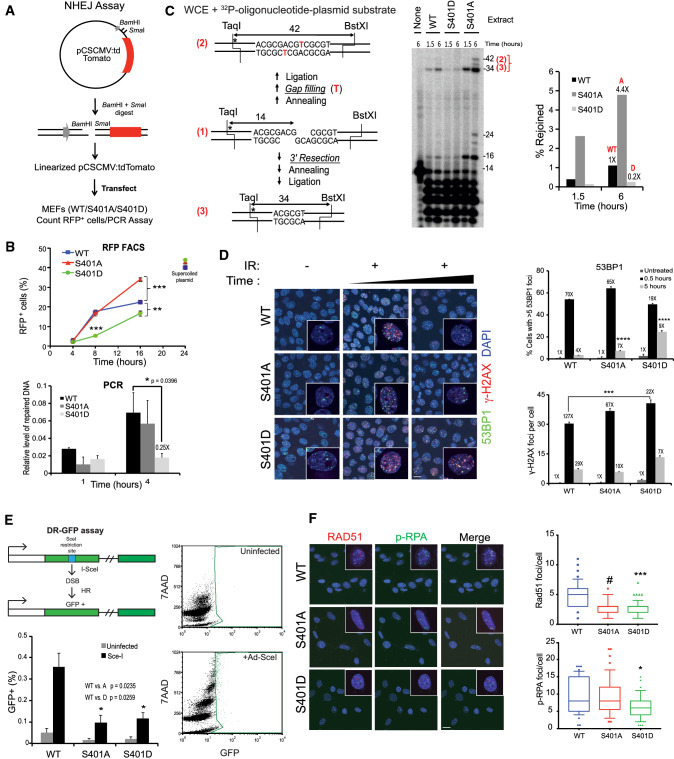
DSB repair is abnormally high in S401A cells while being suppressed in S401D. **A** Scheme for in vivo end joining (EJ) assay. Linearized Tomato expression plasmid was transfected into MEFs followed by quantification of RFP^+^ cells (Nexcelom Cellometer) and PCR. **B** Quantification of EJ by RFP flow cytometry (*top panel*). S401A cells have super-active EJ at the later times after transfection whereas S401D cells have suppressed EJ throughout. qRT-PCR assay was carried out to determine early EJ (bottom panel). The y axis denotes the relative levels of repaired DNA (*n* = 3)*.* EJ in S401D cells was suppressed by 75% at 4 h in line with the results by RFP flow. **C** In vitro end joining. End joining of an internally labeled (*) plasmid substrate with partially complementary ends in MEF extracts (left panel). Following incubation for the indicated times, substrates were cut with BstXI and TaqI and analyzed on sequencing gels (middle panel). Gap-filling products (42-base fragment) and resection-based products (34-base fragment) were quantified. The 24- and 16-base fragments are the corresponding head-to-head end joining products. Graph shows sum of the major 34-base fragments in each case (right panel). **D** Mutant S401 cells show aberrant IR-induced foci with delayed disappearance of 53BP1 and γ-H2AX foci in S401 mutant cells (left panel). MEFs were seeded in a chambered slide. Cells were exposed to 2 Gy of ionizing irradiation and fixed after 0.5 and 5 h. Cells were immuno-stained with anti-53BP1 and anti-γ-H2AX and counterstained with DAPI (blue) to label nuclei. Representative images 53BP1 (green), γ-H2AX (red) and DAPI (blue). Images were acquired on Zeiss LSM 710 confocal microscope at × 63 power. Repair foci remain significantly longer in S401A and -D (esp. D) than in WT cells suggesting aberrant DSB repair. Delayed disappearance of 53BP1 (top, right panel) and γ-H2AX (bottom, right panel) foci in S401 cells post irradiation. Five fields were assessed in at least 2 independent experiments. Error bars: mean ± SEM. Statistical analysis was carried out using unpaired, two-tailed *t* tests. *p* values are shown as ***(*p* < 0.005) is considered significant. Scale bar is 10 μm. **E** S401A and D cells are impaired in homologous recombination using the DR-GFP assay (top, left panel). PR65-DR-GFP (WT, S401A and S401D) cells were infected with Ad-SceI (bottom, right panel) or not (top, right panel) and 72 h later, cell populations analyzed by GFP flow cytometry after incubation in 0.1% Triton-X-100 in PBS. The results (bottom, left panel) are presented as mean ± SEM (30,000 events), *n* = 3, * p < 0.05 relative to WT Scale bar is 10 μm. **F** Reduced Rad51 and pRPA foci in S401 mutants post-CPT treatment. MEFs were treated with 100 nM of CPT for 6 h, fixed and immunostained with anti-pRPA32(S4/S8) and anti-Rad51 antibodies, and counterstained with DAPI (blue). Representative images Rad51 (red), pRPA (green) and DAPI (blue) images acquired on a Zeiss LSM 710 confocal microscope at 63 × power (left panel). Quantification of Rad51 and pRPA32 (S4/8) foci (right panels). Rad51 foci per cell were counted in 5 separate fields in at least two independent experiments. Error bars; mean ± SEM. Statistical analysis was carried out using unpaired, two-tailed *t* tests. *p* values expressed as *(*p* < 0.05), ***(*p* < 0.005), and ^#^(*p* < 0.001) were considered significant. For Rad51 foci, WT vs. S401A; *p* = 0.0008, WT vs. S401D; *p* = 0.0011. For pRPA foci, WT vs. S401D; *p* = 0.0443

In addition to reversing changes in chromatin structure caused by H2AX phosphorylation, PP2A is believed to facilitate NHEJ by dephosphorylating KU70/80 and DNA-PKcs, which are critical participants in NHEJ [14], and for promoting their release from DNA [58]. Furthermore, PP2A restores kinase activity of phosphorylated, inactive DNA-PKcs in vitro to regulate NHEJ [59]. To determine how S401 alterations might affect DSB repair, we then carried out in vivo as well as in vitro end joining assays. First, in a reporter assay, WT, S401A and S401D mutant cells were transfected with a linearized pCSCMV:tdTomato plasmid with incompatible restriction endonuclease DNA ends unable to ligate. Once the plasmid is repaired and circularized, the red fluorescence protein (RFP) expressed is a measure of end joining activity (Fig. 4A). Post transfection, cells were collected at various times and RFP+ cells quantified. At 16 h the S401A cells had almost twice the number of RFP+ cells relative to WT whereas the S401D cells had significantly less than the WT cells throughout (Fig. 4B, top panel). To examine early repair, we quantified the circularized plasmid at 1 and 4 h after transfection and determined end joining yield by quantitative PCR (Fig. 4B, bottom panel). The PCR result is in agreement with what we observed with the reporter assay at early times, i.e., S401D cells have compromised end joining. S401A cells probably did not have enough time to translate RFP into showing elevated repair over WT at 4–8 h that was seen at 16 h by FACS.

To determine the possible effect of PP2A-mediated dephosphorylation on end joining in vitro, a ^32^P-oligonucleotide ligated into a plasmid substrate bearing partially complementary 5′ overhangs was incubated in whole-cell extracts from WT, S401A, and S401D cells for 1.5 or 6 h (Fig. 4C). As in our previous work [60, 61], the predominant 34-base head-to-tail product as well as the 16-base head-to-head product reflects 3′ resection and annealing at a 4-base micro-homology (Fig. 4C, left panel). We observed dramatically reduced end joining activity with the extract from S401D, whereas the S401A extract markedly enhanced it and yielded an additional 42-base product corresponding to gap filling without resection (Fig. 4C, middle and right panels). Thus, while the critical PP2A dephosphorylation targets are currently unknown these results show that using S401 mutants as surrogates for mimicking (de)phosphorylated S401 is important for end joining by reporter as well as in cell-free in vitro assays. In line with these results, S401D cells showed abnormal, extensive and more persistent 53BP1 foci after radiation compared with WT (Fig. 4D, left panel). 53BP1 is critical for directing DSB repair towards NHEJ at the expense of HRR [62]. 53BP1 foci co-localized with γ-H2AX, suggesting that NHEJ is significantly altered and delayed in S401D cells. Furthermore, S401A cells had fewer but more pronounced foci at 5 h than either WT or S401D (Fig. 4D, right panel). Altogether, S401 mutant cells have significantly altered end joining activity in quality as well as temporal execution, yet S401D and S401A differed in that the latter showed super-active end joining whereas S401D was severely compromised.

### S401 mutants have impaired HRR

Since NHEJ and HRR are in competition in S/G2 of the cell cycle and we observed irregular end joining in the S401 mutants, we then examined HRR in the cell panel. The PP2A holoenzyme associated with the B55 subunit is implicated in HRR via the modulation of ATM phosphorylation and the G1/S checkpoint [63], as well as the dephosphorylation of pRPA32 mediated by PP2A after replication fork recovery, which might impact HRR [57]. To determine the importance of the S401 residue in HRR, we generated CKO/CKO MEFs with a chromosomally integrated DR-GFP repair reporter [38], followed by infection with either WT, S401A and S401D retrovirus and floxing of the endogenous PR65 gene with Ad-CMV-Cre (Fig. S6) as before (see Fig. 1B, E). We noticed that HRR was significantly reduced in S401A and S401D cells represented by lower GFP+ events (Fig. 4E). During HRR, single stranded DNA generated during DNA resection is first coated with RPA and then replaced by RAD51 during Holliday junction formation and resolution critical for efficient HRR [14, 64]. Therefore, we treated the cells with CPT and then co-stained for pRPA32 (S4/8) and RAD51 (Fig. 4F). Both S401A and S401D cells had ~ 40% less RAD51 foci compared with WT cells (Fig. 4F, right-top panel) with pRPA also trending lower in S401D relative to WT and S401A (Fig. 4F, right-bottom panel). This finding is in line with the result with the DR-GFP reporter and the conclusion that HRR is impaired in S401A and S401D cells relative to WT. Altogether, it is quite clear from our results so far that S401 mutant cells have rewired DDR and show aberrant DSB repair. Surprisingly, however, these mutants display fairly minor effects on cell survival after radiation and CPT treatment. This is particularly striking with the S401D cells since they are compromised in end joining as well as HRR.

### Increased aberrant chromosomal DSB repair and translocation in S401 mutants

**Fig. 5:**
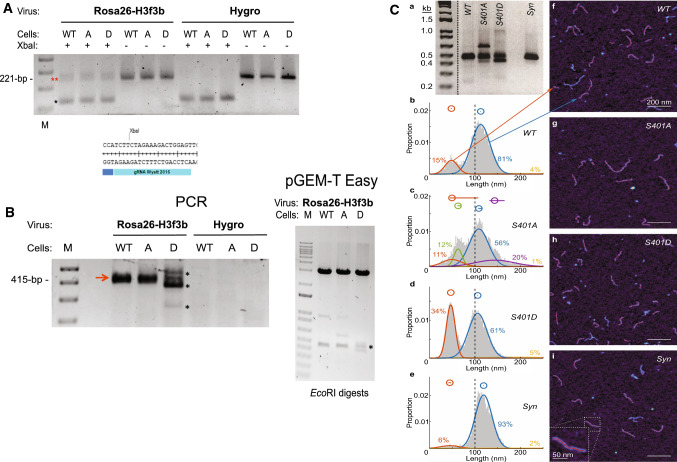
CRISPR-Cas9 mediated DSB repair. **A** DSB repair at the Rosa26 (Chr 6) locus. XbaI digestion removes background PCR DNA without indels (no NHEJ)* and leaves behind the ~ 221-bp PCR fragments with indels (red **) (*top panel*). Primers: Chr6 Rosa26-F/Rosa26-R [67]. The Rosa26 CRISPR-Cas9 gRNA target spans an XbaI restriction site (*bottom panel*). Thus, an indel would eliminate the XbaI site. **B** Rosa26 (Chr 6) and H3f3b (Chr 11) translocation between DSBs generated by dual CRISPR-Cas9 cleavage. Primers: Chr6 Rosa26-F/Chr11 H3f3b-R [67]. Major indels at translocation junctions are seen only in D cells* although translocation is seen in all three cell mixes (left panel). PCR fragments were cloned into pGEM-T Easy and sequenced (right panel). **C** Fragment size distributions of Rosa26-H3f3b translocation amplification products as determined by **a** 2% agarose gel electrophoresis and **b**–**i** high-speed atomic force microscopy (HS-AFM). Four samples were amplified: wild type (WT, *n* = 11,303 measured strands), S401A (*n* = 6530), S401D (*n* = 12,160), and a synthetic fragment (Syn, *n* = 6035) of equivalent length and sequence as WT. In **b**–**e**, the normal probability density of amplicon lengths for each sample is shown as well as a Gaussian mixture model fit to each distribution. The probability density function of each single Gaussian fit is shown as a smooth colored curve, with that curve's population proportion given in the same color. Also, above the probability density plots, the mean of each Gaussian fit is plotted as a hollow circle, and the 95% confidence interval of the mean is shown as a horizontal line (CI calculated from a bootstrap simulation with *n* = 10,000) (Supplementary Table S1). In **f**–**i**, high-speed AFM images of DNA amplicons from each sample are shown with 200 nm scale bars and lighter colors representing greater height. In frame **b**, arrows point from the two main distributions to imaged strands in frame **f** that belong to the indicated population. In frame **i**, a higher magnification of a single strand is shown. The red backbones shown on some amplicons represent the measured lengths of those amplicons; if an amplicon has no red backbone, it did not meet the image analysis algorithm's quality standard and was not measured

Alternative end joining (Alt-EJ, now referred to as Theta-mediated end joining; TMEJ) and other micro-homology repair pathways are believed to become engaged when c-NHEJ (classical end joining) and/or HRR are not fully functional or being disengaged [65–67]. However, TMEJ is highly mutagenic because of the promiscuous activity imposed by DNA polymerase Theta (Polθ)-mediated chromosomal repair and rearrangements through short (2–4 bp) stretches of micro-homology in resected DNA ends. To assess TMEJ in our PR65 cell panel we examined DSB repair quality after CRISPR/Cas9-mediated DNA cleavage at the Rosa26 locus and by chromosomal translocation between the Rosa26 and H3f3b loci on Chr. 6 and 11, respectively [66, 67]. The fact that the Rosa26 cleavage site is positioned in the middle of an XbaI site allows for the elimination of any DNA resealing products without indels, which if present would destroy the XbaI site, as well as remove DNA that was not cut by CRISPR/Cas9 (Fig. 5). As expected, XbaI-resistant PCR fragments remained after cleavage with Cas9–Rosa26/H3f3b gRNAs expressed but not without when examining events happening at the Rosa26 site (Fig. 5A). Cells not expressing Cas9–Rosa26/H3f3b gRNAs (*Hygro*) did not have any XbaI-resistant DNA as expected.

Because of the complexity of analysis and difficulties achieving meaningful statistical support for differences in the quality and quantity of end joining products between cells in the panel at the single Rosa26 DSB, we decided to examine the spectrum of translocations occurring between Chr. 6 and 11 after inducing dual chromosomal breaks [66, 67]. TMEJ appears to play an important role in chromosomal translocations in human cells [68–71]. However, in the mouse, the role of Pol Theta in translocation repair is more controversial [72]. Nevertheless, with PCR primers amplifying translocation events as an expected 415-bp fragment, we found that the S401D cells produced relatively large insertions as well as deletions compared with either WT or S401A cells which gave bands closer to the expected PCR fragment size without large indels (Fig. 5B, left panel). The PCR fragments were cloned and some sequenced (Fig. 5B, right panel; Fig. S7). Among the DNA sequences, we found indels with micro-homology in the DNA from S401D and S401A cells that seemed more extensive and with larger inserts than from WT. Altogether, translocations generated in S401D cells are to a large extent associated with significant microhomology end joining since predominant number of DNA sequences had short 2–3 bp repeats in the junction between Chr. 6 and 11. In line with the results generated throughout this study using different DNA repair assays, we again noticed differences in the quality of DSB repair between the three cell populations.

### Assessing translocation events by atomic force microscopy

Because cloning of PCR fragments might be biased against isolating longer inserts many of which might have direct or inverted repeats (that could be unstable in bacteria), we decided to utilize a novel, high-throughput technique that does not require plasmid cloning. A recently developed alternative for determining translocations by indel size is using atomic force microscopy (AFM) [73]. High-speed AFM-based physical mapping of DNA allows single-molecule measurement of thousands of PCR products in a relatively short time. However, only the sizes of the indels are determined without establishing the DNA sequence but the assay is nevertheless a way to quantify differences in DNA repair quality between the three cell populations, simplify the process, and, importantly, allow for better statistical analysis. In this experiment, we noticed that S401A as well as S401D generated translocation indels seen on an agarose gel whereas few were seen from WT cells or a synthetic (*Syn*) template without any indel at the Rosa26–H3f3b junction that served as standard (Fig. 5C, panel a). When the AFM results were examined in more detail, we noticed shifts in the size of indels between the three cell populations (Fig. 5C, panels b–e) and images of the DNA corresponding to the various curves (Fig. 5C, panels f–i). The *Syn* template produced sizes centered at 119 nm (blue; 93%) and a bulge of smaller sizes around 45 nm (orange; 6%) representing background noise products (panels e, i, Supplementary Table S1). WT cells predominantly gave sizes around 113 nm (blue; 81%) slightly smaller than with the *Syn* standard template, indicating smaller deletions at the junction as expected as well as smaller fragments around 48 nm (orange; 15%) some of which possibly represent deletions of 200-bp or more (panels b, f). The size pattern resulting from S401A was more complex (panels c, g), with a main peak at 108 nm (blue; 56%), a broad peak centered at 143 nm and extending > 260 nm (purple; 20%) in size, and a range of deletions with peaks between 49 and 61 nm (green; 12%, orange; 11%). Finally, S401D produced a predominant peak centered around 106 nm (blue; 61%), slightly smaller in size then either WT or S401A and an abundant peak centered around 47 nm (orange; 34%) (panels d, h). The overall pattern and differences in fragment size distribution of indels seen between WT, S401A, and S401D by AFM are significant (Supplementary Table S1). The observed distinct peaks might represent repair events resulting from preferred micro-homology hotspots such as repetitive DNA in resected ends that might serve as sinks for repair. Clearly, the quality of DSB repair is dramatically altered when S401 is replaced with either a null or a phospho-mimetic substitution reflecting the importance of PR65 S401 (de)phosphorylation during the DDR. However, to determine the mechanism behind these differences and whether they represent TMEJ or other (backup) DSB repair goes beyond the scope of this report and will have to wait for a more thorough future investigation.

### S401 (de)phosphorylation shuttles PR65 between the nucleus and the cytoplasm

**Fig. 6:**
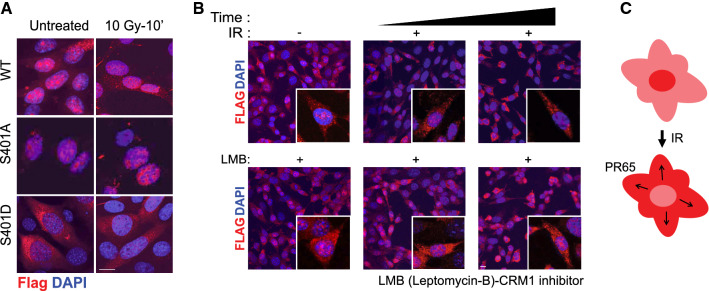
Radiation-induced nuclear export of WT PR65 is mimicked by S401D. **A** Wild-type MEFs grown in chamber slides were irradiated with 10 Gy and fixed at 10 min after irradiation then stained with anti-Flag antibody to detect PR65 (red) and counterstained with DAPI (blue). Images were acquired on Zeiss LSM 710 confocal microscope at 40 × power. **B** Irradiation results in cytoplasmic accumulation of Flag-PR65 seen best at 60 min (top panels. LMB blocks PR65 nuclear–cytoplasmic shuttling as shown by the increased nuclear and decreased cytoplasmic accumulation at 30–60 min (bottom panels. MEFs were grown on chamber slides, treated or not with leptomycin B (LMB) present (1 ng/ml; at -3 h) in the medium. Cells were irradiated with 10 Gy or not, and fixed at 30 and 60 min post-IR. Scale bar is 10 μm. **C** Model for radiation-induced nuclear export of PR65 mediated by S401 phosphorylation (see text)

To return to the overarching goal of this study and how growth, cell cycle regulation, and DSB repair could be so fundamentally altered by a single amino acid change mimicking the phosphorylation or not of PR65 at S401 by the ATM kinase, we wanted to explore this issue more thoroughly. In neurons, ATM phosphorylates PR65 at S401 resulting in nuclear–cytoplasmic shuttling of HDAC4 and PR65 [31]. Thus, we examined the cellular location of WT, S401A and S401D PR65 in the MEFs using Flag antibody. In WT cells PR65 was present in the cytoplasm as well as the nuclear compartment whereas S401A was predominantly nuclear and in S401D exclusively cytoplasmic with little change after radiation except that in the WT and S401A cells PR65 appeared more in punctate nuclear foci (Fig. 6A). It is unlikely that all PR65 complexes participate in a unison manner blurring the pattern and the interpretation thereof. This observation in addition to the sequestration of S401D mutant in the cytoplasm suggests that the phosphorylation of PR65 at S401 causes it to translocate from the nucleus to the cytoplasm in line with a previous report [31]. To explore the mechanism in more detail, we treated WT cells with radiation pretreated or not with leptomycin B (LMB), which is a very specific inhibitor of the nuclear export protein CRM1 (chromosome region maintenance 1) responsible for the nuclear export of many proteins and RNAs [74]. After radiation, PR65 accumulated in the cytoplasm in a temporal fashion which was prevented by LMB resulting in more nuclear PR65 (Fig. 6B, compare middle and right panels ± LMB). These results suggest that the nuclear export of PR65 after radiation and phosphorylation by ATM kinase is mediated by CRM1 (Fig. 6C).

### PR65 shuttles to the cytoplasm by CRM1 via a nuclear export sequence

**Fig. 7:**
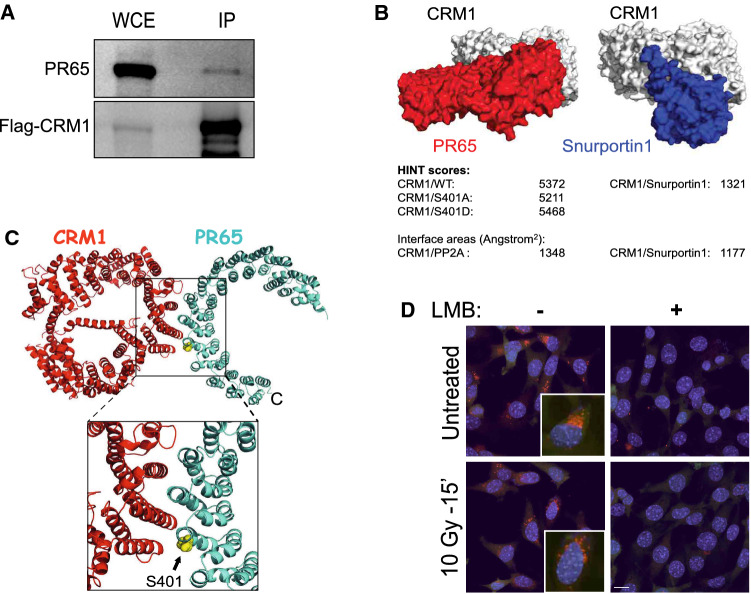
PR65 and CRM1 physically interact. **A** CRM1 forms a complex with PR65 as shown by co-immunoprecipitation with Flag-CRM1. HEK293 cells were transfected with Flag-CRM1, mRuby2-PR65, and RanQ69L expression plasmids followed by whole-cell immunoprecipitation at 72 h using Flag-beads and western blotting with anti-PR65 and -Flag antibodies. **B** HINT analysis of CRM1-PR65 and CRM1-Snurportin 1 interactions suggest that CRM1-PR65 hydrophobic interaction is stronger and has a larger interface area than between CRM1 and Snurportin 1. **C** Modeling suggests that CRM1 (red) interacts with the -L_365_LPLFLAQL_373_- motif of PR65 (cyan) with S401 in the vicinity of the binding interface (yellow sphere). **D** PR65 and CRM1 co-localize at the cytoplasmic–nuclear membrane interface. Proximity Ligation Assay (PLA) shows that PR65 and CRM1 interact within 30–40 nm prior to irradiation with 10 Gy. After irradiation, this interaction seemed more punctuated at the cytoplasmic–nuclear membrane. Pretreatment with LMB (1 ng/ml) eliminates any sign of PR65–CRM1 interaction Scale bar is 10 μm

After DNA damage, several DDR proteins including p53, BRCA1, and BRCA2 are exported out of the nucleus through NES-mediated CRM1 processes [75–77]. To determine whether PR65 might have a NES, we scanned the PR65 amino acid sequence for putative hydrophobic leucine residue motifs. We identified two leucine residues, L370 and L373, that scored positive as potential residues of a NES sequence located between PR65 amino acids 365–373 (LLPLFL_370_AQL_373_) which resembles the consensus NES, LxxxLxxLxL [78] (Fig. S8A). Nuclear export of proteins harboring a functional NES sequence requires CRM1 binding to the target [79–81]. Therefore, we examined whether CRM1 and PR65 interact by performing co-immunoprecipitation (IP) of lysates from HEK293 cells expressing Flag–CRM1 and mRuby2–PR65 also including a plasmid expressing the RanQ69L mutant that is incapable of exchanging GTP for GDP and prevent the dissociation of the Ran–CRM1 complex in whole cell extracts [82, 83]. The IP/western blotting experiment shows that CRM1 interacts with PR65 (Fig. 7A).

One representative example of a CRM1 interactive partner is Snurportin-1 (Exportin-1) for which a co-crystal structure has been determined (PDB ID 3GB8). This structure was then used for identifying possible CRM1–PR65 hydrophobic interactions by HINT analysis modeling hydrophobic protein interactions under aqueous condition [84–86]. We found that the docked CRM1–PR65 model has a HINT score of more than fourfold higher than CRM1-Snurportin-1 that is reduced when WT is replaced with S401A and increased with S401D (Fig. 7B). In addition, the CRM1–PR65 interface area was significantly larger compared with CRM1–Snurportin-1. Furthermore, modeling suggests an interaction between CRM1–K537 and the -L_365_LPLFLAQL_373_-motif of PR65 with S401 positioned in the vicinity of the binding interface (Fig. 7C, Fig. S8B). Upon S401 phosphorylation, a tighter interaction is expected between K537 and pS401, or alternatively S401D, because of increased electrostatic interaction (Fig. S8B). S401 is close to the surface of PR65 in the PP2A holoenzyme and at the interface where PR65 interacts with the PP2A catalytic (C) subunit between HEAT repeat 10–15 (Fig. S8C, right panel). Interestingly, the putative PR65 NES is located in the inter-repeat loop between HEAT domains 10 and 11 and in close proximity to S401 within HEAT repeat 10 [87], and close to the outside boundary of the PP2A holoenzyme possibly more accessible for phosphorylation by ATM kinase (Fig. S8C, left panel).

To confirm that PR65 and CRM1 interact in the cell, we carried out a proximity ligation assay (PLA). PR65–CRM1 proximity was observed as a fluorescent signal in untreated and irradiated WT cells with more punctate perinuclear appearance after irradiation (Fig. 7D). As expected, the PR65–CRM1 interaction was disrupted when cells were pretreated with LMB. Altogether, these results suggest that PR65 is exported to the cytoplasm by CRM1 after S401 is phosphorylated.

**Fig. 8:**
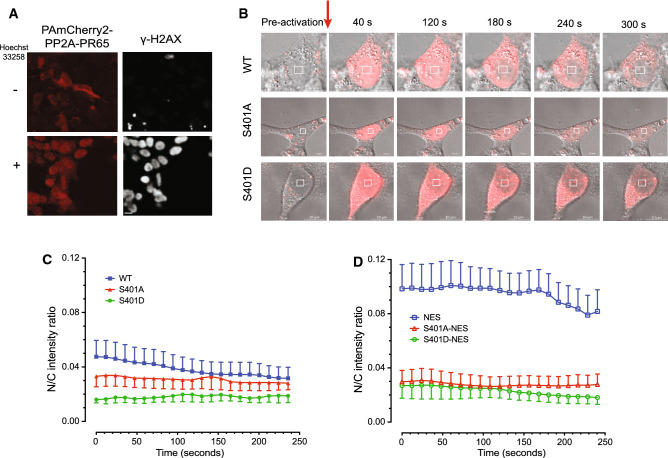
DNA damage triggers PR65 movement from the nucleus to the cytoplasm. **A** UV-A photoactivation of nuclear PR65 in HEK293 cells after adding Hoechst 33258 (+) or not (*−*) results in increased DNA damage and DSBs as indicated by the increased γ-H2AX nuclear staining. Scale bar is 10 μm. **B** pPAmCherry-PR65-WT is exported to the cytoplasm after nuclear irradiation. HEK293 cells were transfected with pPAmCherry-PR65 (WT, S401A and S401D) in glass bottom dishes and 48 h post transfection subnuclear ROI (4 μm × 4 μm), marked by the squares, were photoactivated by 405 nm laser (242.8 μJ) and followed over time (≤ 300 s) by live cell imaging using a Zeiss LSM 710 microscope with images acquired at × 63. WT-PR65 is exported from nucleus to cytoplasm over time whereas PR65–S401A is retained in the nucleus and PR65–S401D remains cytoplasmic. Scale bar is 10 μm. **C** PR65-S401 mutants show impaired ability to shuttle between nucleus and cytoplasm. Transfected WT (*n* = 28), S401A (*n* = 42) and the S401D (*n* = 22) cells were photoactivated and followed over time (≤ 240 s). In WT cells the N/C was as high as 0.05 initially and declined over several minutes, suggesting that PAmCherry–PR65 was nuclear early after DNA damage and quickly shuttled to the cytoplasm. On the other hand, both S401A and S401D cells showed N/C ratios that were initially 30–60% less (0.015–0.03) and remained the same throughout the experiment, suggesting that PR65–S401A and -S401D were statically nuclear and cytoplasmic, respectively (left panel). **D** When HEK293 cells were transfected with pPAmCherry–PR65 WT/L373A (*n* = 11) and photo-activated, the initial N/C fluorescence intensity ratio was 0.10, twice the ratio seen with WT, and the signal did not decline over time except at  > 170 s that might represent photo bleaching. The S401A (*n* = 23) and S401D (*n* = 10) mutants were less affected by the L373A addition, but, interestingly, the flat N/C ratios seem to come more together compared with S401 alteration alone (right panel). An one-way ANOVA was performed on the time series. *p* < 0.0001 for WT vs S401A, WT vs S401D, NES vs NES-S401A, and NES vs NES-S401D

Because PP2A is such an abundant protein constituting ~ 1% of total protein in a mammalian cell and involved with regulating many processes throughout the cell it is technically challenging to follow a PR65 subpopulation engaged in one specific process out of many in the cell using immunostaining or standard fluorescence protein fusions because of preexisting background. Therefore, we transfected photoactivatable plasmid constructs pPAmCherry-PR65 WT, S401A, and S401D into HEK293 cells and demonstrate similar expression levels as endogenous PR65 (Fig. S9). Laser photo-activation of a specific subcellular location such as the nucleus would allow for following the shuttling of PR65 in live cells after DNA damage with less interference from unrelated PR65 events. Forty-eight hours post transfection a subnuclear region-of-interest (ROI) was irradiated with a 405 nm laser to photo-activate PAmCherry-PR65 WT. We determined that UV-A alone was sufficient to cause DNA damage without including Hoechst 33528 to the medium based on the γ-H2AX and phospho-KAP1 (S824) foci formation although the addition of dye increased DNA damage (Fig. 8A, Fig. S10). We then confirmed our previous results with Flag-immunofluorescence (see Fig. 6), now using PAmCherry as reporter, that PR65-WT was exported from the nucleus to the cytoplasm whereas S401A was not, and PR65-S401D remained in the cytoplasm (Fig. 8B, Fig. S11, Supplementary videos 1–3). We plotted the nuclear to cytoplasmic ratios (N/C) from the collected fluorescence signals. With WT cells, the N/C was as high as 0.05 initially and declined over several minutes (Fig. 8C, Supplementary Table S2), suggesting that PAmCherry–PR65 was nuclear early after photo-activation resulting in DNA damage and then quickly shuttled to the cytoplasm. On the other hand, the S401A and S401D N/C ratios were initially 30–60% less (0.015–0.03) and remained the same throughout the experiment, suggesting that PR65–S401A and -S401D were statically nuclear or cytoplasmic, respectively (Fig. 8C, left panel). These results are consistent with the notion that nuclear PR65 is exported to the cytoplasm after DNA damage in a pS401-dependent manner. In addition, in support of the physical presence of mutant PR65 in the nucleus or cytoplasm, presumably with altered PP2A substrate specificity, is the distinct pattern of survival of MEFs overexpressing different B regulatory subunits after transfection with plasmids (Fig. S12). We found a similar pattern of tolerability when B55α, B56α, B56β, B56δ, and B56γ3 were overexpressed in WT and S401 mutant cells, whereas a dramatically varied response was seen with B56γ1 and B56ε, suggesting that PR65 has preferential B subunit partners depending on S401 phosphorylation status and location.

To determine whether the putative PR65 NES sequence functions, we generated cells stably transfected with photoactivatable plasmid constructs, pPAmCherry–PR65 WT, S401A, and S401D, also having the L373A alteration. When HEK293 cells were transfected with pPAmCherry–PR65 WT/L373A and then photo-activated, the initial N/C fluorescence intensity ratio was 0.10, twice the ratio seen with WT (Fig. 8C), and the signal did not decline over time except at > 170 s that might represent photo bleaching (Fig. 8C, right panel, Supplementary Table S2). The S401A and S401D mutants were less affected by the L373A addition, but, interestingly, the flat N/C ratios seem to come more together initially compared with either S401 alteration alone. Altogether, these results suggest that the ATM kinase modulates the early stages of the DDR by phosphorylating PR65 at S401 thereby exposing a buried NES to CRM1 and resulting in PR65 nuclear export. In doing so, physically removing PR65 is expected to reduce or alter PP2A activity in the nucleus.

## Discussion

PP2A plays a major role in numerous cellular processes, including regulating the DDR and DNA repair [25, 27, 29, 30]. ATM kinase phosphorylates PR65 at S401 and when mutated is associated with dysfunctional neuronal chromatin deacetylation by HDAC4 resulting in possible neurodegeneration in ataxia telangiectasia [31]. Previous work by our group explored how DSB signaling through ATM converges on prosurvival signaling through AKT [21]. Negative regulation of AKT by PP2A is well established [88, 89]. Herein we report that phosphorylation of a single, critical amino acid residue (S401) in the PR65 subunit is important for DNA damage signaling, DNA repair, and cell survival recovery.

Our molecular modeling simulations suggest that the phosphorylation of S401 causes the horseshoe-shaped scaffolding subunit PR65 to undergo a conformational change. This leads to the disassociation of the ATM–PR65 complex, as previously reported to occur after radiation [25], which, herein is supported by our co-IP results of mutant PR65 forms. We show that ATM, AKT, and PP2A-C no longer interact with S401-phosphorylated PR65 or the analogous phospho-mimetic mutant. Impaired interaction of S401D with PP2A-C could therefore fundamentally alter PP2A phosphatase activity, substrate targeting, and/or cellular localization. A reciprocal IP/western blot experiment might resolve whether AKT is part of the PR65–ATM complex or is a separate PR65–AKT complex.

Interestingly, PR65 and ATM both have many HEAT repeats, 15 and 49, respectively. HEAT repeats consist of pairs of interacting, antiparallel helices linked by flexible “intra-unit” loops believed to form dynamic structures and elastic connectors that link force and catalysis [87, 90]. Additionally, CRM1 has 19 HEAT repeats known to participate in the formation of a nuclear export complex [91]. It is tempting to speculate that these HEAT repeats are involved in a dynamic “hand-over and exchange” process swapping partners during the DDR and resulting in nuclear export of PR65 expected to quickly alter the nuclear phospho-protein landscape and maintain a state of elevated phosphorylation in the nucleus immediately following DNA damage. Presumably, PR65 then shuttles back into the nucleus to turn off the DDR by dephosphorylating proteins targeted directly or indirectly by ATM phosphorylation. Clearly, this is a simplified scenario since several other S/T protein phosphatases such as PP1, PP4 and PP6 have also been linked to the DDR [92]. However, it is well established that these phosphatases perform redundant functions by acting either as the primary phosphatase or secondary backup to avoid cellular crises to occur (e.g., during mitosis).

We, and others, have previously reported that an ATM-dependent phosphatase negatively controls AKT (S473) dephosphorylation in response to insulin and radiation [21, 93]. At the time, we hypothesized that ATM kinase negatively acts on an OA-sensitive phosphatase that in turn dephosphorylates pAKT in the presence of an ATM kinase inhibitor [21]. In the present study, we found that AKT phosphorylation was elevated in S401D mutants relative to the WT and S401A counterparts. However, in response to radiation, the increase in AKT (S473) phosphorylation was impaired in S401D as well as S401A cells. We know that the DDR initially takes place primarily in the nucleus, whereas insulin signaling initiates at the plasma membrane through activation of the insulin receptor and with AKT present in both compartments. It is likely that AKT is differentially regulated in the nucleus and cytoplasm by PP2A. This could be due to reduced PP2A activity and/or inappropriate cellular location of PP2A impairing the normal execution of the DDR. That the response to insulin and radiation are different might be expected because radiation likely produce more pleiotropic effects on signaling than insulin [45]. This may be through association with different B subunits, and as demonstrated herein, in a coordinated PP2A nuclear–cytoplasmic shuttling process to spatiotemporally regulate the DDR as well as cell growth recovery. PP2A, and more specifically the (de)phosphorylation of PR65 at S401, supported by the results presented could be the mechanism by which ATM–PP2A regulates AKT signaling.

γ-H2AX is present at DSBs in the nucleus. From our PR65 localization study, we observed that S401D was retained in the cytoplasm in cells exposed to radiation whereas the WT, and to a lesser extent S401A, was found in both the cytoplasm and nucleus. Thus, cytoplasmic sequestration of PR65–S401D possibly makes cells impaired in nuclear γ-H2AX dephosphorylation. We have also shown that S401D cells are less efficient in DSB repair, accumulate chromosomal abnormalities and show reduced survival after DNA damage. On the other hand, S401A cells have much greater end joining activity compared to WT cells in vitro*,* which is in line with the idea that DNA repair cannot shut off. We propose this is because PR65 is unable to be exported out of the nucleus when S401 cannot be phosphorylated. Furthermore, since PP2A is essential for the radiation G2/M checkpoint [29], the decreased ability of PR65 to shuttle and retain proper PP2A activity in a strict spatiotemporal manner would likely result in checkpoint failure and mitotic catastrophe.

Most notably, both in vitro and in vivo studies suggest that DSB repair is fundamentally altered in the S401 mutants. Future studies will determine in more detail what type of DSB repair is engaged when both c-NHEJ and HRR are compromised as is the case in S401D cells. Likely, a micro-homology type of DSB repair such as TMEJ or SSA is involved to maintain relatively normal cell survival based on our early analyses presented here.

**Fig. 9:**
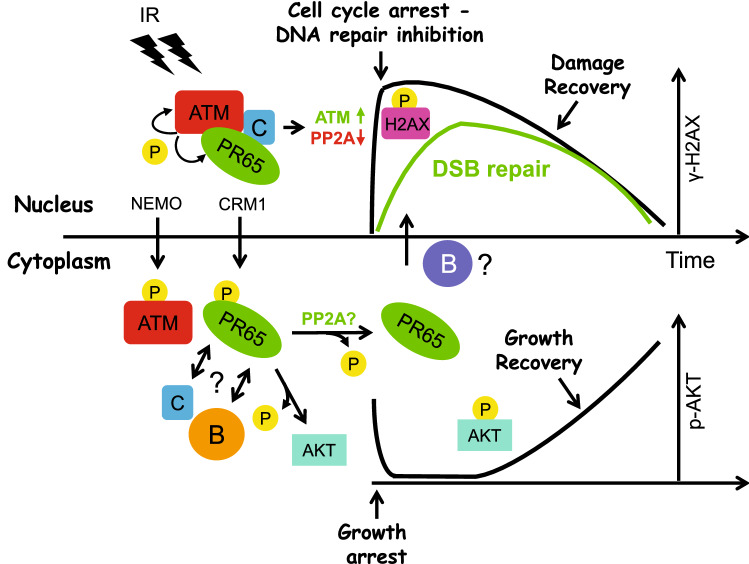
Model for ATM-PP2A mediated regulation of the DDR and cell growth—spatiotemporal (de)phosphorylation and nuclear–cytoplasmic shuttling by ATM and PP2A controls the DDR “ON–OFF” switch. ATM phosphorylates numerous proteins in response to radiation including H2AX at S139 and PR65 at S401 thereby initiating the DDR and triggering cell cycle checkpoints and growth arrest, and temporarily block DSB repair to assess the damage before proceeding. Within minutes, phosphorylated PR65 translocates to the cytoplasm by CRM1 where it then may form a complex with (a) different B subunit(s) and substrate specificity (see Supplementary Fig. 12). Immediately thereafter, ATM is exported to the cytoplasm in a NEMO-mediated process. In the cytoplasm, AKT and other growth-promoting factors and signaling pathways are inhibited (dephosphorylated) by the increased/altered PP2A activity while the DDR subsides. Subsequently, PR65 shuttles back to the nucleus, re-establishes nuclear PP2A activity and dephosphorylate DDR targets allowing the DDR to run its course and bring back the cell to normalcy. When repair is completed and cells resume cycling, growth recovers

As to the mechanism how PR65 S401 alterations might affect cell growth and DNA repair in a reciprocal manner, we propose a model in which PP2A together with ATM might serve as gatekeeper of the DDR (Fig. 9). For ATM-directed activation of the DDR to occur through a phosphorylation cascade, the phosphatase, in this case PP2A, must initially be inactivated or sequestered to maintain a phosphorylated protein landscape. Subsequently, phosphatases have to be reactivated to remove phosphates in order to allow for cell cycle reentry and resumption of growth. If the DNA damage is sublethal, DNA repair is mostly completed within a relatively short time window of 6–12 h [94]. Interestingly, ATM and PP2A form an autoregulatory, yin yang/kinase-phosphatase relationship serving as a potential on–off switch of the DDR. Based on our findings and by others, PP2A is likely having the upper hand in this relationship since PP2A inhibitors activate the ATM kinase, resulting in a pseudo-DDR response without significant DNA damage [25]. There are many examples of this regulatory hierarchy in kinase-phosphatase signaling pathways [95]. Nuclear export seems a fast, efficient means of temporarily removing PP2A from the nucleus and sites of DNA repair. Once repair is complete, PP2A can shuttle back to the nucleus and remove protein phosphates and reset the cell for baseline growth and activity. Therefore, PP2A (along with other phosphatases) must coordinate and balance DNA repair and cell survival with apoptosis resulting from insurmountable cellular damage. Our model incorporates the ATM–PP2A “on–off” switch for the DDR coupled with cell recovery and growth by ATM and PP2A shuttling between the nucleus and cytoplasm and, together, acting as a cellular balancing rheostat for DNA damage and growth control.

Our results suggest that the improper localization of PP2A/PR65 and, thus, untimely dephosphorylation of critical phosphoproteins result in a shift in the quality of DSB repair though without dramatic impact on survival. The most straightforward interpretation of our results is that DSB repair is re-wired in a manner that substitute c-NHEJ and HRR with some other homology-based DNA repair to maintain viability after DNA damage. Regardless, because the only difference between the three cell populations used in our study is the PR65 S401 alterations, this cell system could be a rich source for dissecting DNA repair and growth responses regulated by PP2A that would otherwise not be possible with general DNA damaging treatments, which would trigger a plethora of difficult-to-dissect cellular responses.

## Supplementary Information

Below is the link to the electronic supplementary material.

Supplementary file1 (JPG 616 KB)

Supplementary file2 (PNG 2364 KB)

Supplementary file3 (PNG 2380 KB)

Supplementary file4 (PNG 2777 KB)

Supplementary file5 (PNG 3211 KB)

Supplementary file6 (PNG 2757 KB)

Supplementary file7 (PNG 1230 KB)

Supplementary file8 (XLSX 10 KB)

Supplementary file9 (XLSX 10 KB)

Supplementary file10 (PNG 1287 KB)

Supplementary file11 (PNG 1458 KB)

Supplementary file12 (XLSX 37 KB)

Supplementary file13 (XLSX 13 KB)

Supplementary file14 (XLSX 290 KB)

Supplementary file15 (PPTX 4428 KB)

Supplementary file16 (PDF 19849 KB)

Supplementary file17 (PDF 190 KB)

Supplementary file18 (MP4 614 KB)

Supplementary file19 (MP4 545 KB)

Supplementary file20 (MP4 683 KB)

## Data Availability

All the data supporting the findings of this study are either available within the paper and its Supplementary Information files or can be obtained from the authors upon request. Data Source files have been submitted.

## Declarations

### Reporting summary

Further information on research design is available in the Nature Research Reporting Summary linked to this article.
