# Supplementary figures and images for: ATM phosphorylates PP2A subunit A resulting in nuclear export and spatiotemporal regulation of the DNA damage response
Source: Cell Mol Life Sci. 2022 Nov 24;79(12):603. doi: 10.1007/s00018-022-04550-5 (PMC9700600; doi:10.1007/s00018-022-04550-5)

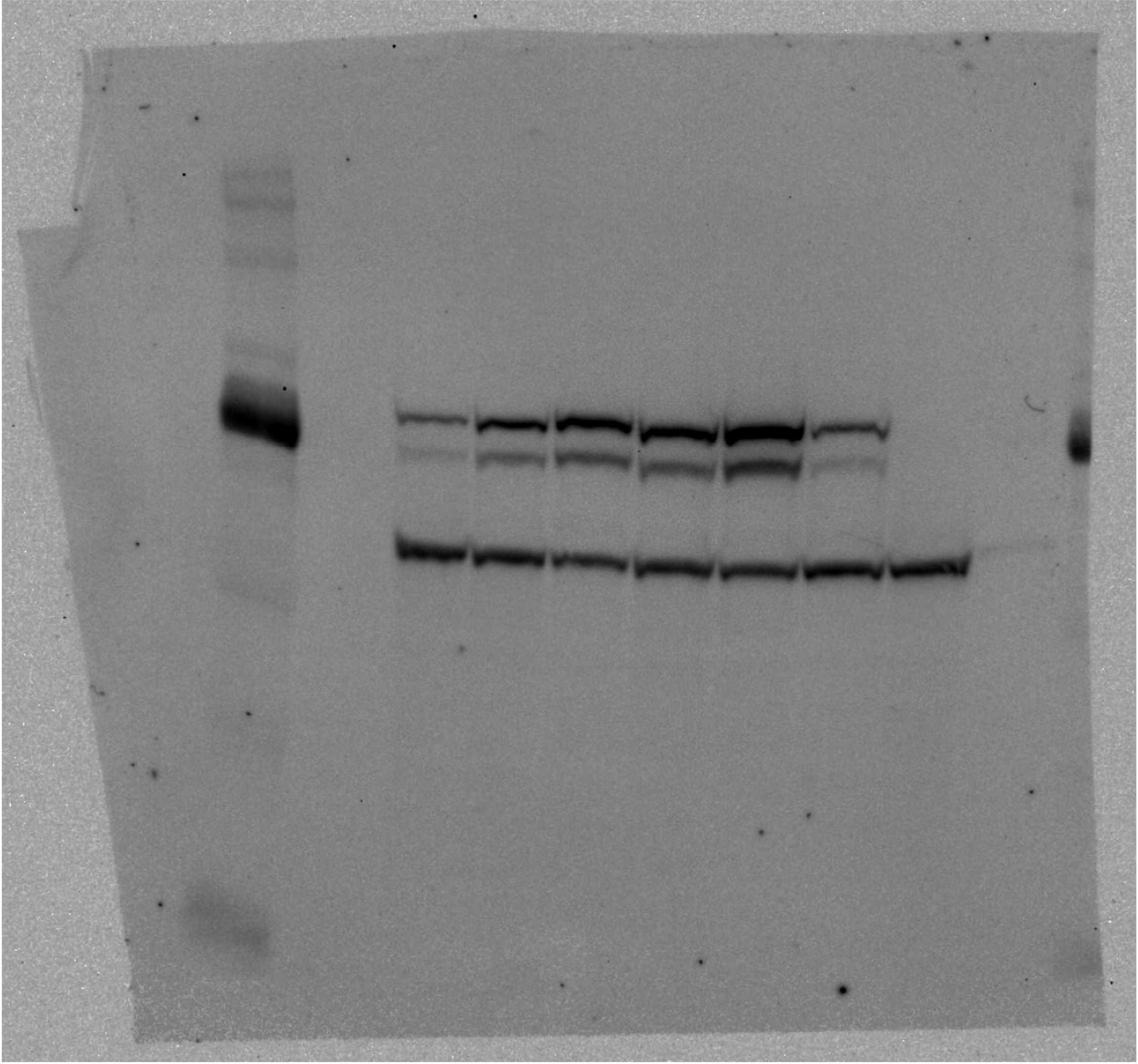

Supplement: Supplementary file 1 — Supplementary file1 (JPG 616 KB) [file 18_2022_4550_MOESM1_ESM.jpg]

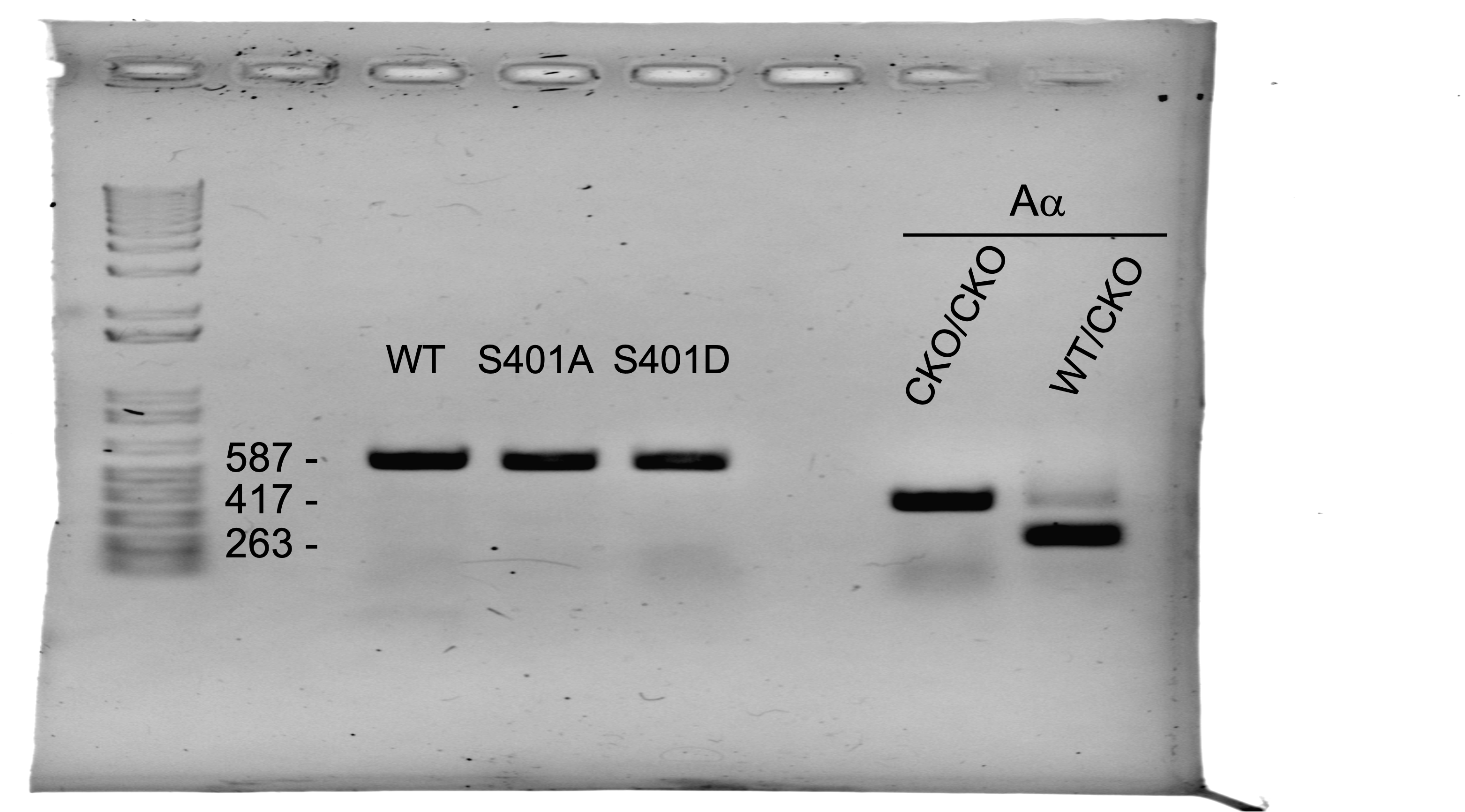

Supplement: Supplementary file 2 — Supplementary file2 (PNG 2364 KB) [file 18_2022_4550_MOESM2_ESM.png]

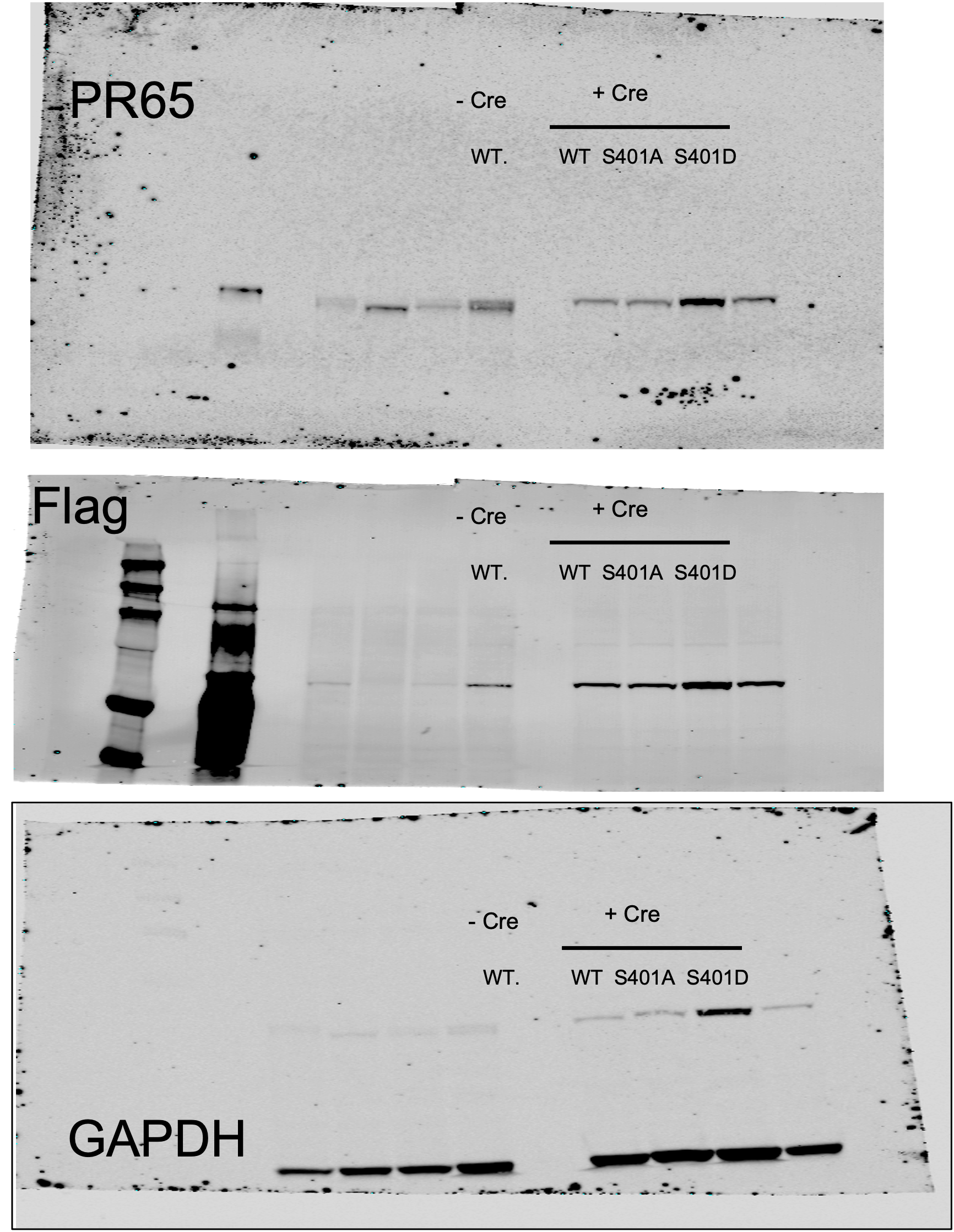

Supplement: Supplementary file 3 — Supplementary file3 (PNG 2380 KB) [file 18_2022_4550_MOESM3_ESM.png]

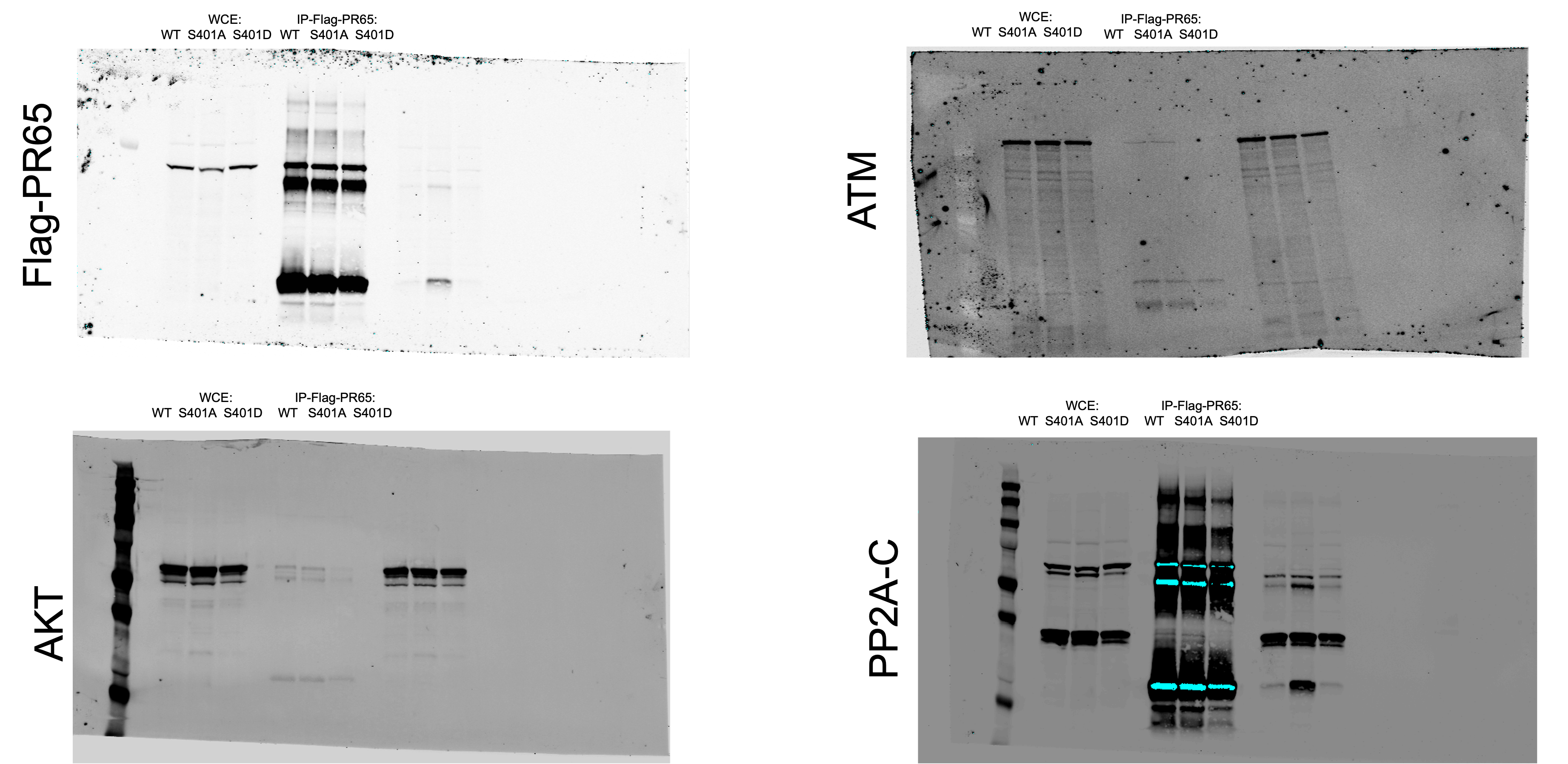

Supplement: Supplementary file 4 — Supplementary file4 (PNG 2777 KB) [file 18_2022_4550_MOESM4_ESM.png]

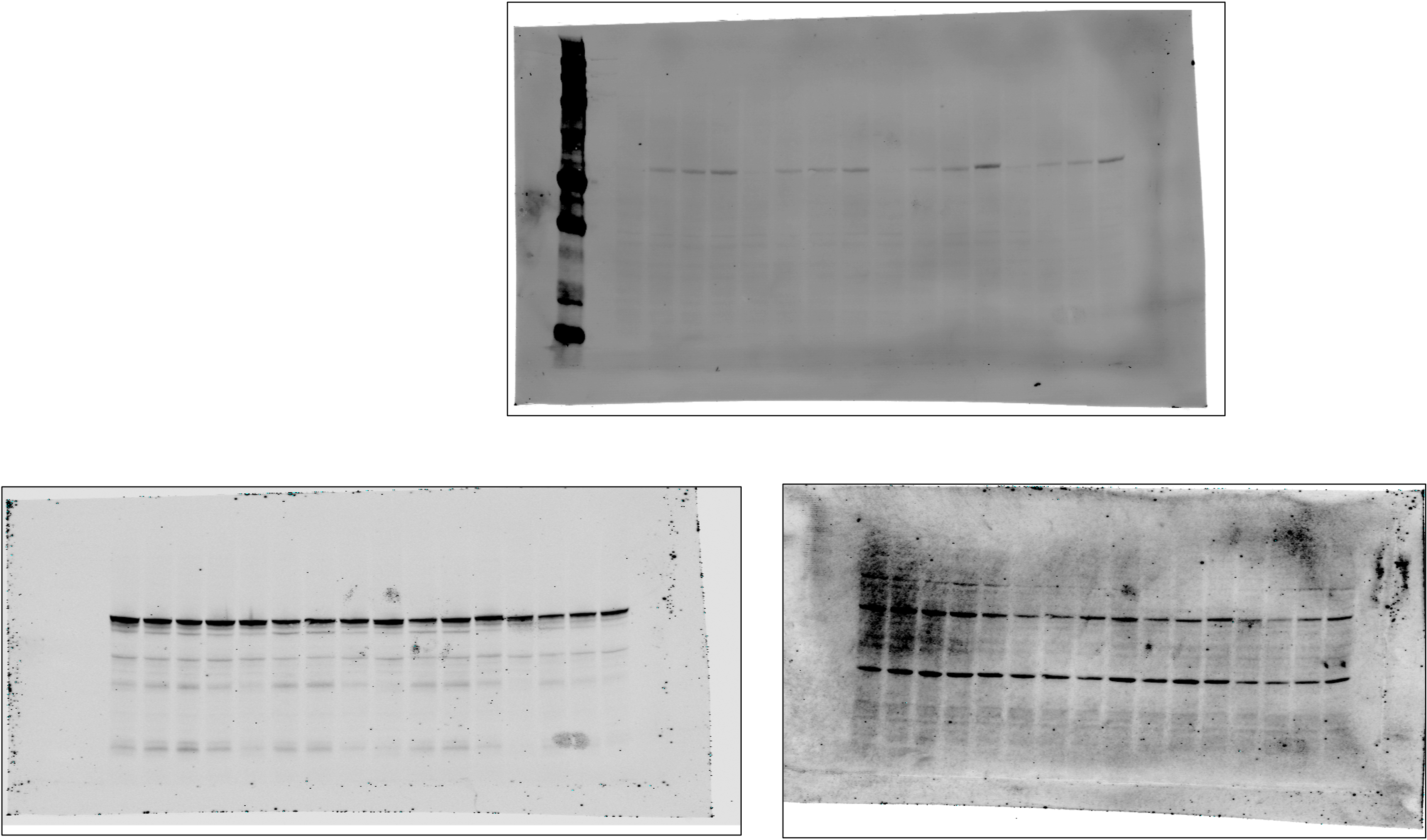

Supplement: Supplementary file 5 — Supplementary file5 (PNG 3211 KB) [file 18_2022_4550_MOESM5_ESM.png]

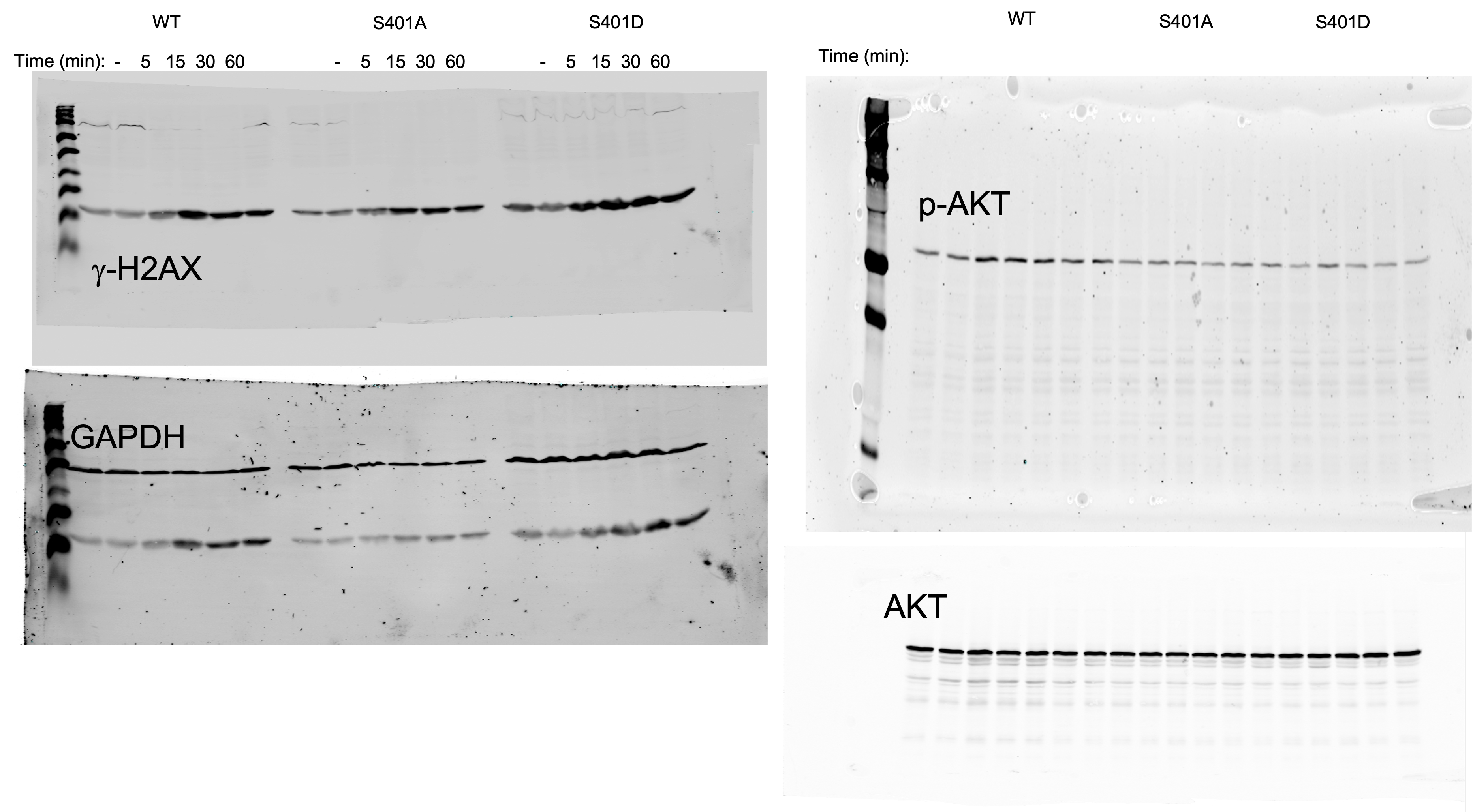

Supplement: Supplementary file 6 — Supplementary file6 (PNG 2757 KB) [file 18_2022_4550_MOESM6_ESM.png]

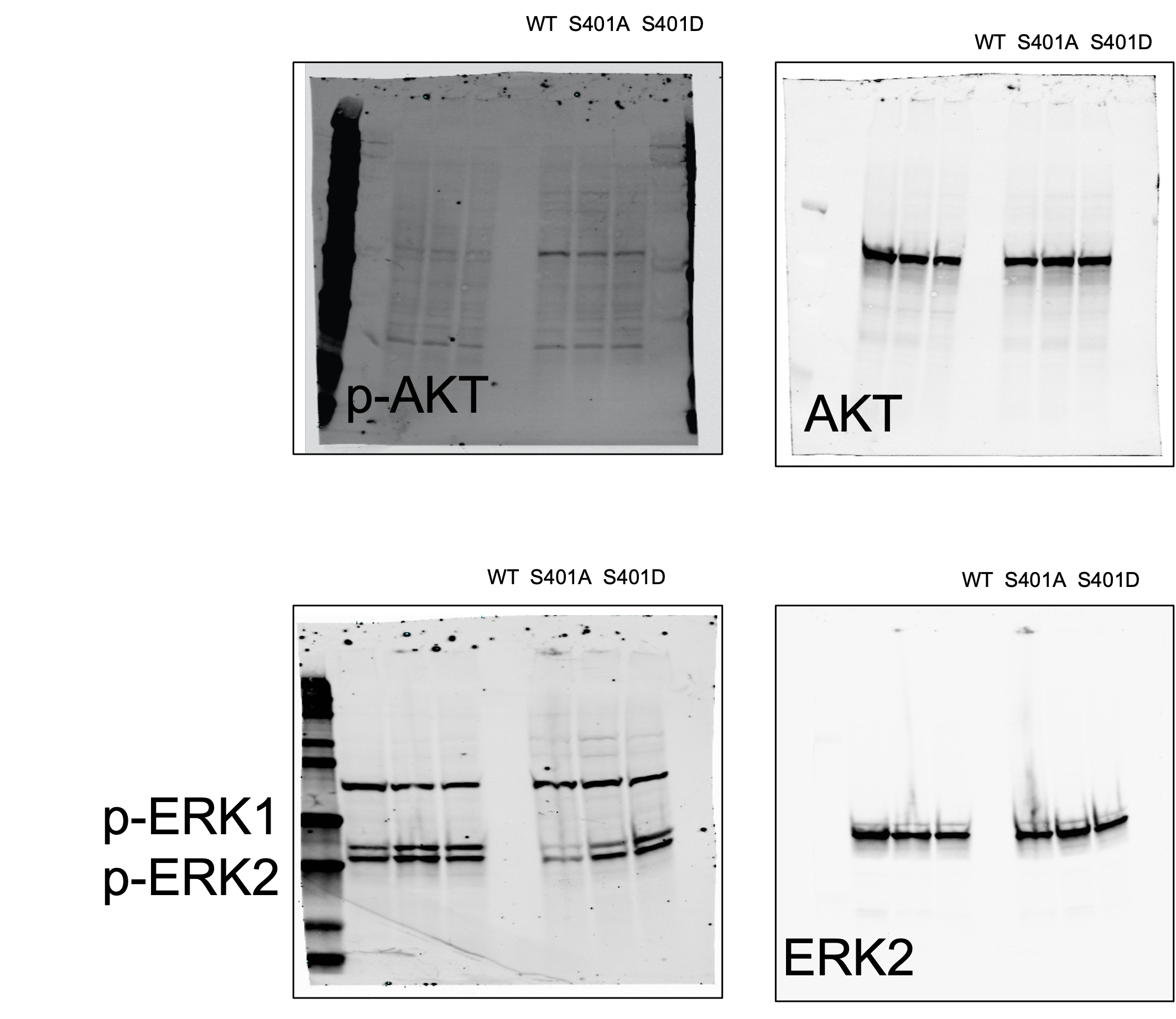

Supplement: Supplementary file 7 — Supplementary file7 (PNG 1230 KB) [file 18_2022_4550_MOESM7_ESM.png]

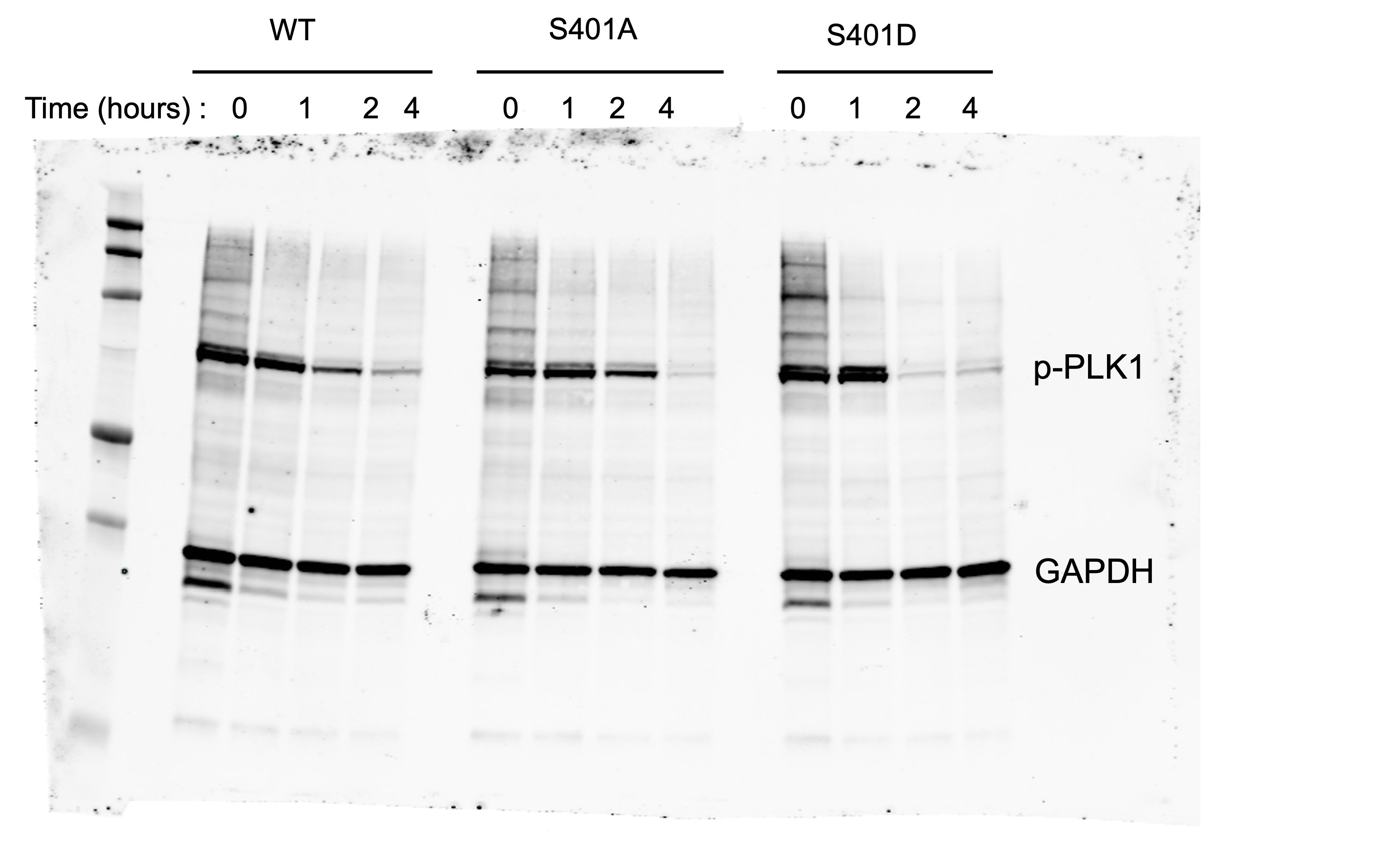

Supplement: Supplementary file 10 — Supplementary file10 (PNG 1287 KB) [file 18_2022_4550_MOESM10_ESM.png]

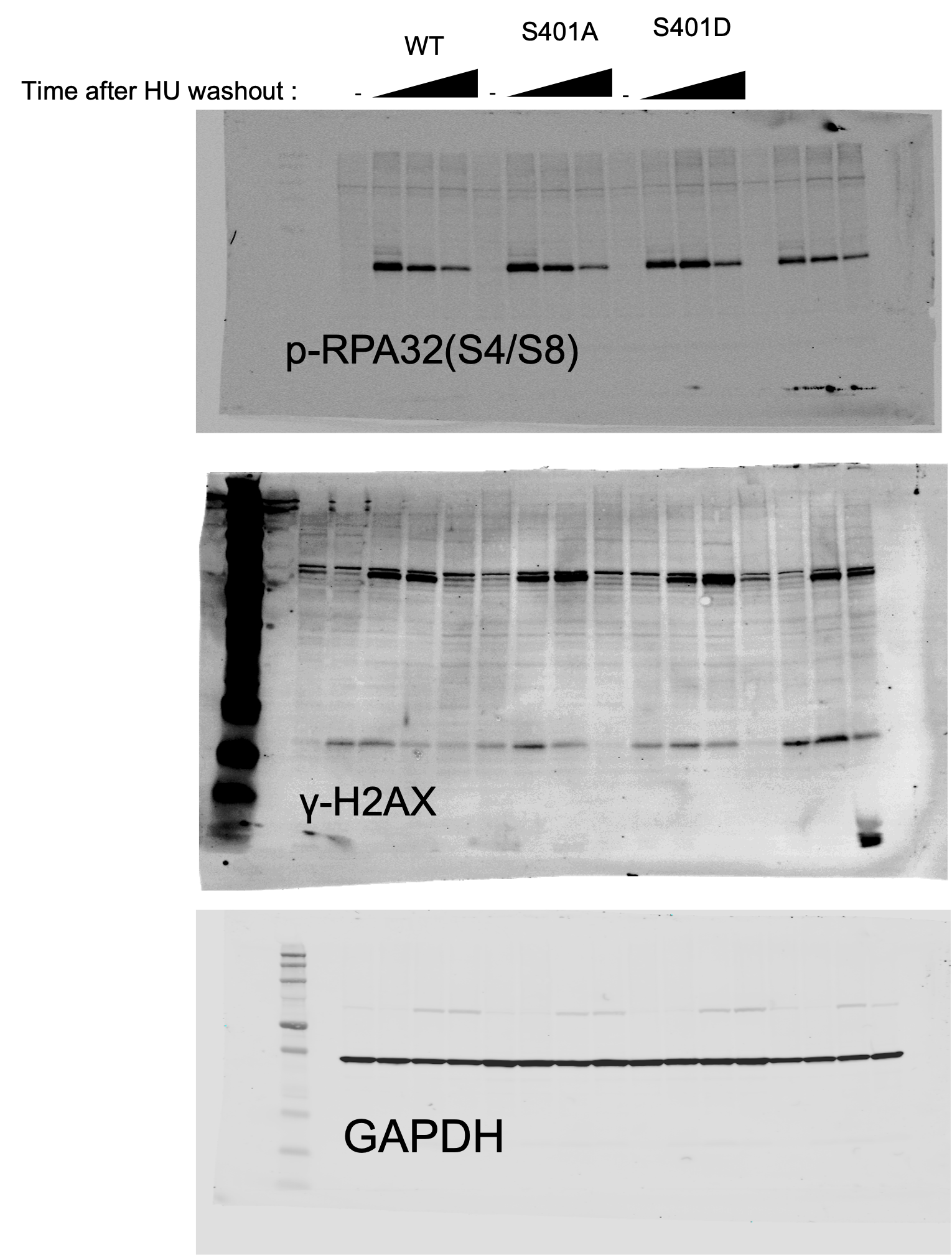

Supplement: Supplementary file 11 — Supplementary file11 (PNG 1458 KB) [file 18_2022_4550_MOESM11_ESM.png]
