# Supplementary material for: ATM phosphorylates PP2A subunit A resulting in nuclear export and spatiotemporal regulation of the DNA damage response
Source: Cell Mol Life Sci. 2022 Nov 24;79(12):603. doi: 10.1007/s00018-022-04550-5 (PMC9700600; doi:10.1007/s00018-022-04550-5)
Supplement: Supplementary file 15 — Supplementary file15 (PPTX 4428 KB) [file 18_2022_4550_MOESM15_ESM.pptx]

## Slide 1
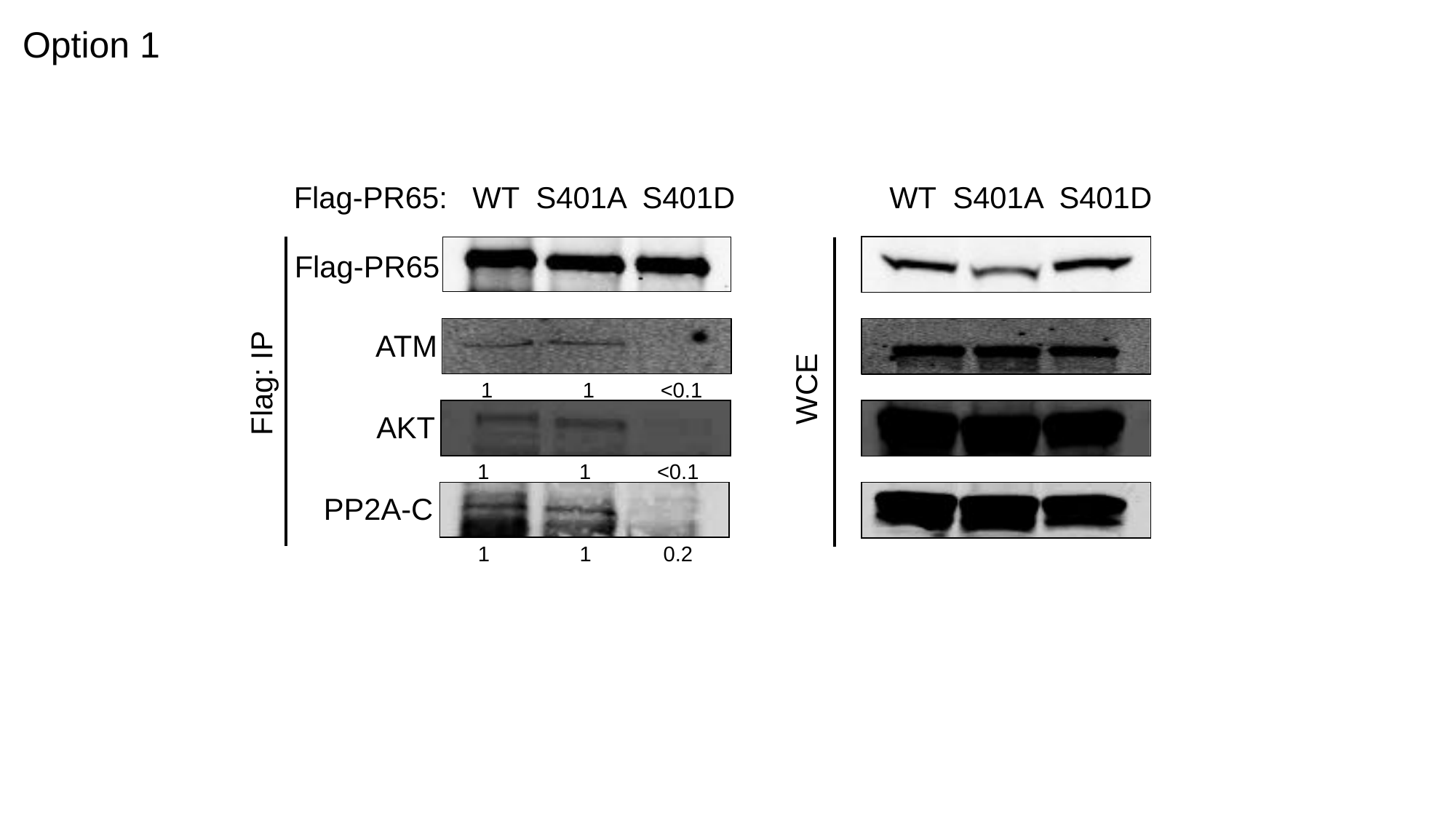

Option 1
Flag-PR65: WT S401A S401D
 WT S401A S401D
Flag-PR65
ATM
Flag: IP
WCE
 1 1 <0.1
AKT
 1 1 <0.1
PP2A-C
 1 1 0.2

## Slide 2
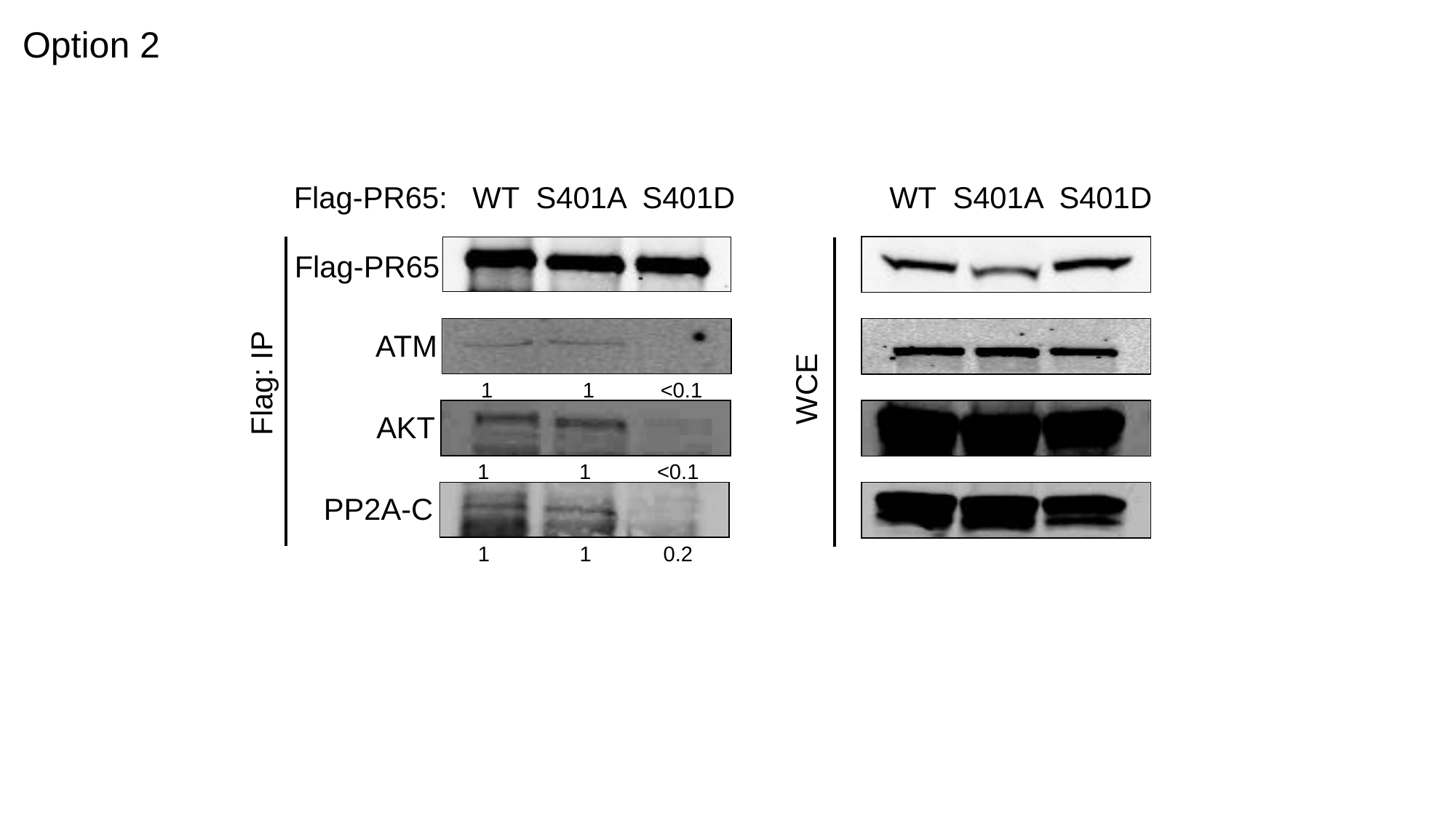

Option 2
Flag-PR65: WT S401A S401D
 WT S401A S401D
Flag-PR65
ATM
Flag: IP
WCE
 1 1 <0.1
AKT
 1 1 <0.1
PP2A-C
 1 1 0.2

## Slide 3
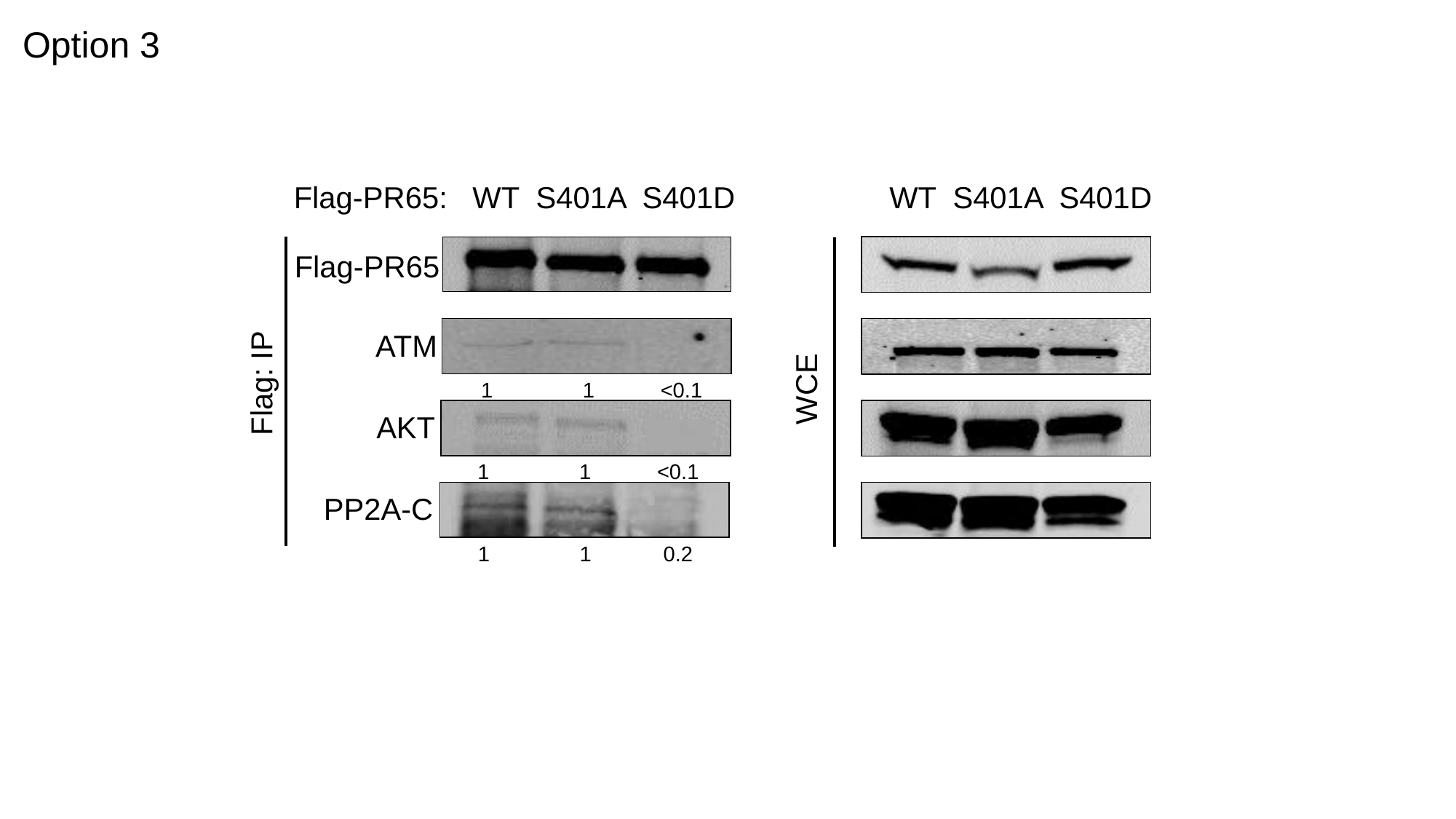

Option 3
Flag-PR65: WT S401A S401D
 WT S401A S401D
Flag-PR65
ATM
Flag: IP
WCE
 1 1 <0.1
AKT
 1 1 <0.1
PP2A-C
 1 1 0.2
