# Supplementary material for: ATM phosphorylates PP2A subunit A resulting in nuclear export and spatiotemporal regulation of the DNA damage response
Source: Cell Mol Life Sci. 2022 Nov 24;79(12):603. doi: 10.1007/s00018-022-04550-5 (PMC9700600; doi:10.1007/s00018-022-04550-5)
Supplement: Supplementary file 16 — Supplementary file16 (PDF 19849 KB) [file 18_2022_4550_MOESM16_ESM.pdf]

Supplementary Figure 1.

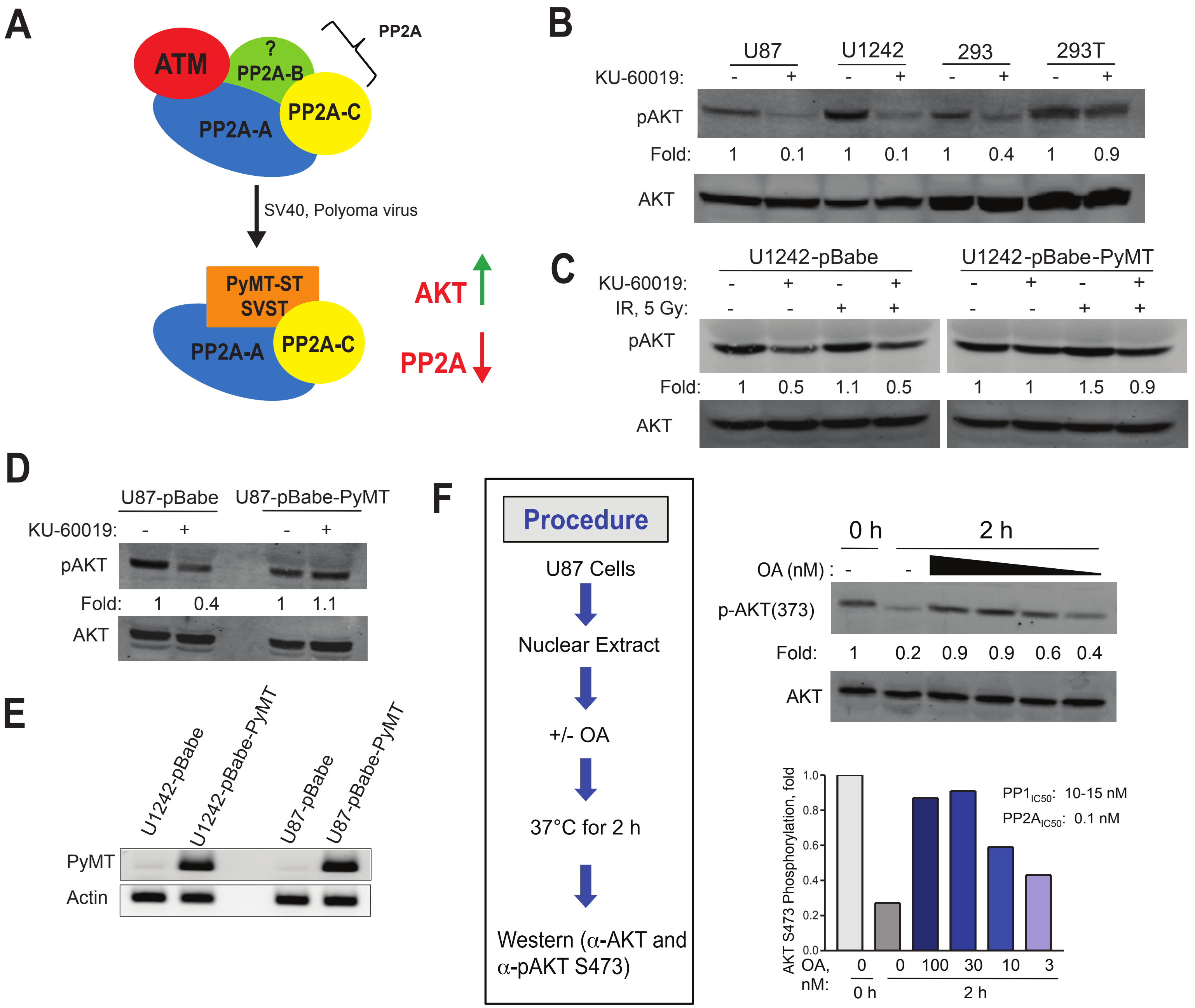

Supplementary Figure 2.

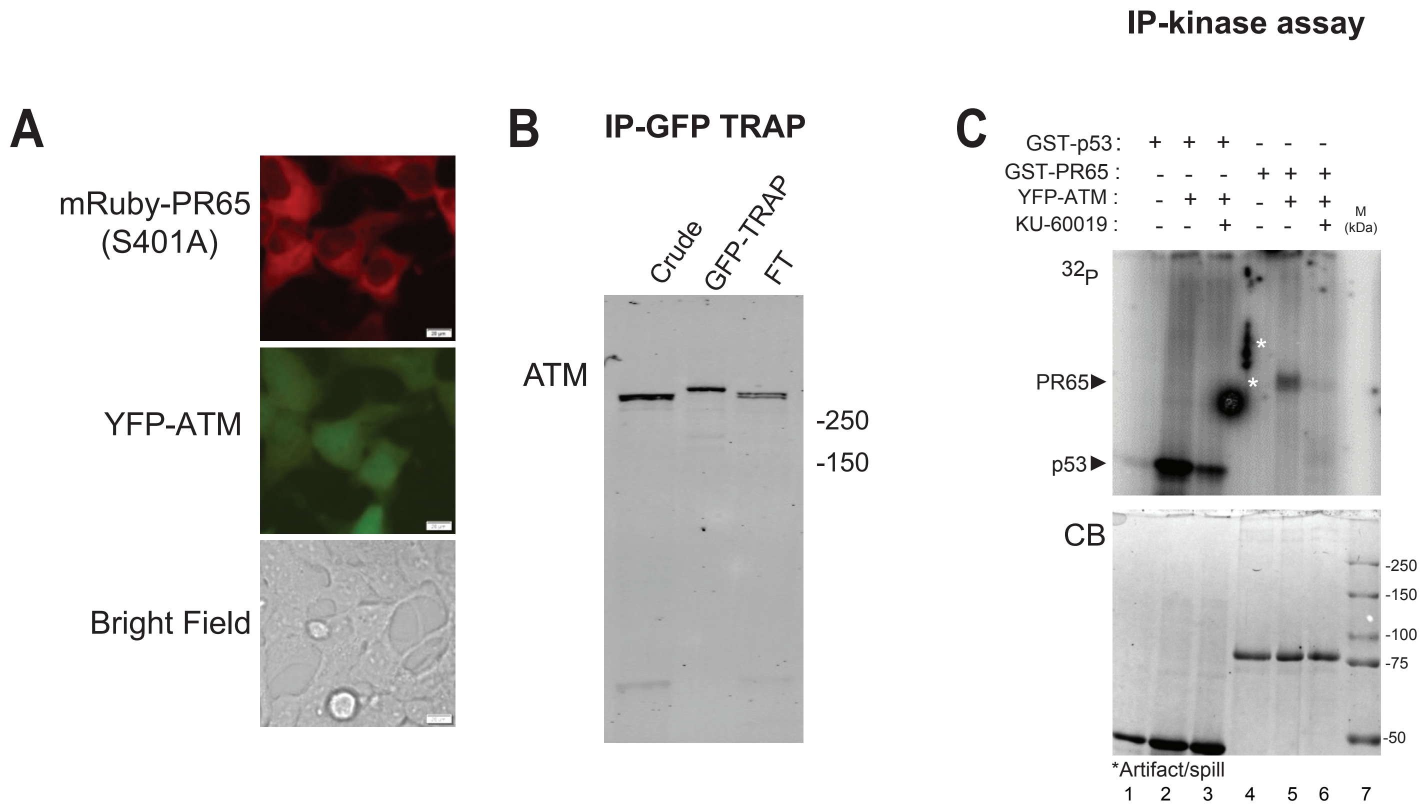

Supplementary Figure 3.

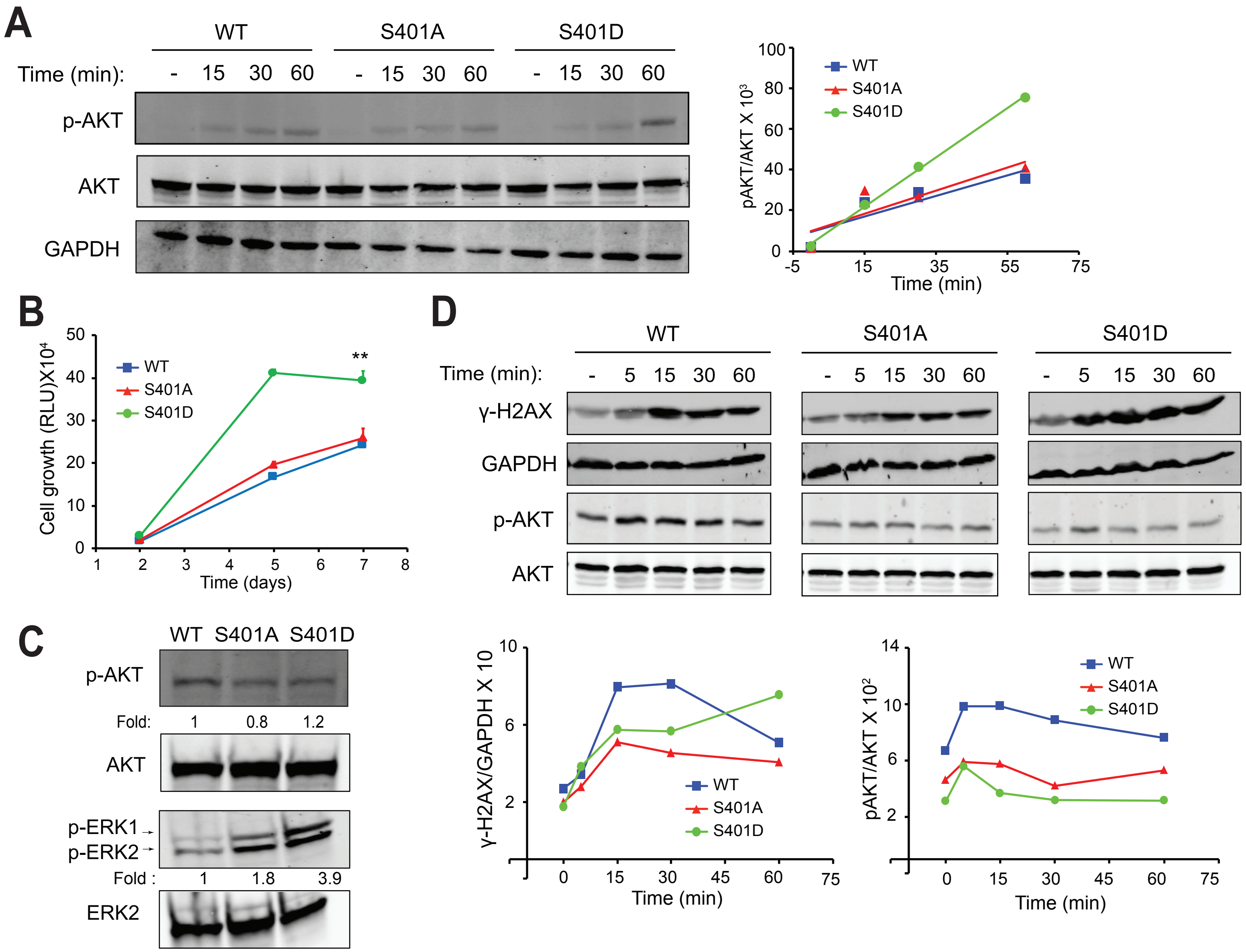

Supplementary Figure 4.

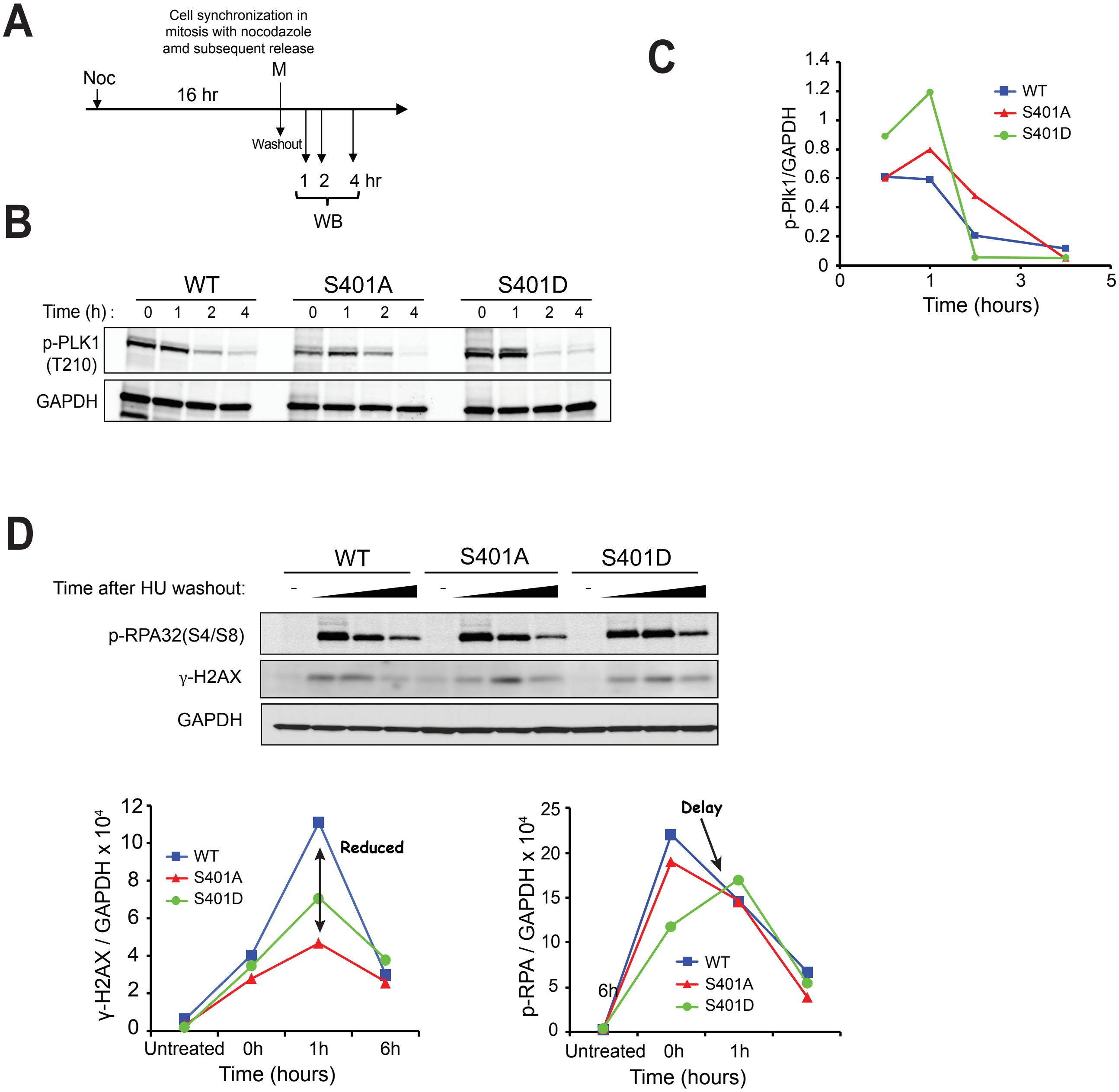

Supplementary Figure 5.

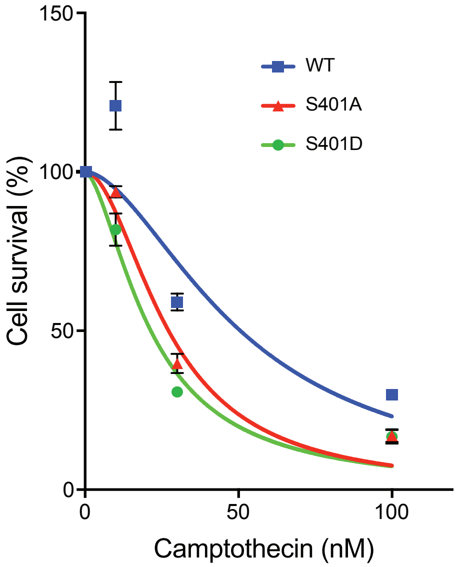

Cells	WT	S401A	S401D
IC50 (nM)	50.61	27.22	21.43

Supplementary Figure 6.

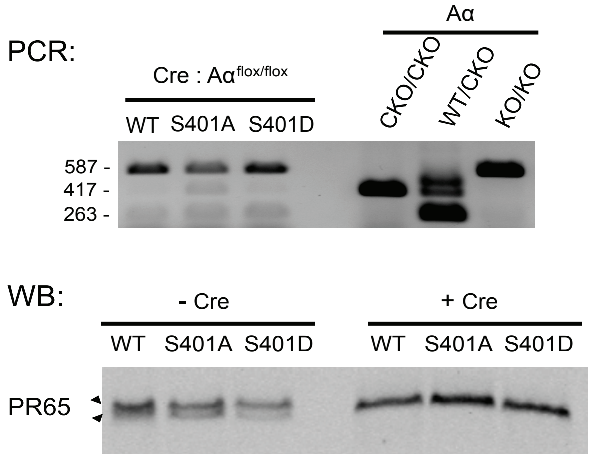

Supplementary Figure 7.

MEFs expressing PR65 WT

Chr-11		Chr-6
ATGCCATAAAAACCGCTTCAACTTAAGCTCTCTCCCCCCGTATCCGGCGAGCC		TCTAGAAAGACTGGAGTTGCAGATCACGAGGGAAGAGGGGGAAGGGATTCT
ATGCCATAAAAACCGCTTCAACTTAAGCTCTCTCCCCCC		TCTAGAAAGACTGGAGTTGCAGATCACGAGGGAAGAGGGGGAAGGGATTCT
		TCTAGAAAGACTGGAGTTGCAGATCACGAGGGAAGAGGGGGAAGGGATTCT
ATGCCATAAAAACCGCTTCAACTTAAGCTCTCTCCCCCC		TCTAGAAAGACTGGAGTTGCAGATCACGAGGGAAGAGGGGGAAGGGATTCT
CTGCCCTCCTCTCCGCTCCAGATTAGCCTCTCTCCCCCC		TCTAGAATTACTGGAGTTGCGGATCTCGAGGGAAGAGGGGGAAGGGATTCT
ATGCCTTAAAAACCTCTTCAACTTAAGCTCTCTCCCCCC		TCTATAAAGACTGGAGTTGCTGATCACGAGGGAANAGGGGGAAGGGTTTCT
ATGCCATAAAAACCGCTTCAACTTAAGCTCTCTC		GGAGTTGCAGATCACGAGGGAAGAGGGGGAAGGGATTCT
ATGCCATAAAAACCGCTTCAACTTAAGC TCTCTC		GGAGTTGCAGATCACGAGGGAAGAGGGGGAAGGGATTCT
ATGCCATAAAAACCGCTTCAACTTAAGCTCTCTCCCCCC		TCTAGAAAGACTGGAGTTGCAGATCACGAGGGAAGAGGGGGAAGGGATTCT
ATGCCATAAAAACCGCTTCAACTTAAGCTCTCTCCCCCCG	G	TCTAGAAAGACTGGAGTTGCAGATCACGAGGGAAGAGGGGGAAGGGATTCT
ATGCCATAAAAACCGCCTCAACTTAAGCTCTCTCCCCCC		GTTGCAGATCACGAGGGAAGAGGGGGAAGGGATTCT
ATGCCATAAAAACCGCCTCAACTTAAGCTCTCTCCCCCC		GTTGCAGATCACGAGGGAAGAGGGGGAAGGGATTCT
ATGCCATAAAAACCGCTTCAACTTAAGCTCTCTCCCCCC	CG	TCTAGAAAGACTGGAGTTGCAGATCACGAGGGAAGAGGGGGAAGGGATTCT
ATGCCATAAAAACCGCTTCAACTTAAGCTCTCTCCCCCC	CG	TCTAGAAAGACTGGAGTTGCAGATCACGAGGGAAGAGGGGGAAGGGATTCT
ATGCCATAAAAACCGCCTCAACTTAAGCTCTCTCCCCCC		GTTGCAGATCACGAGGGAAGAGGGGGAAGGGATTCT
ATGCCATAAAAACCGCTTCAACTTAAGCTCTCTCCCCCCG	G	TCTAGAAAGACTGGAGTTGCAGATCACGAGGGAAGAGGGGGAAGGGATTCT
ATGCCATAAAAACCGCTTCAACTTAAGCTCTCTCCCCCC	CG	TCTAGAAAGACTGGAGTTGCAGATCACGAGGGAAGAGGGGGAAGGGATTCT
ATGCCATAAAAACCGCTTCAACTTAAGCTCTCTCCCCCC	CG	TCTAGAAAGACTGGAGTTGCAGATCACGAGGGAAGAGGGGGAAGGGATTCT
ATGCCATAAAAACCGCCTCAACTTAAGCTCTCTCCCCCC		GTTGCAGATCACGAGGGAAGAGGGGGAAGGGATTCT
ATGCCATAAAAACCGCTTCAACTTAAGCTCTCTCCCCCCG	G	TCTAGAAAGACTGGAGTTGCAGATCACGAGGGAAGAGGGGGAAGGGATTCT
ATGCCATAAAAACCGCCTCAACTTAAGCTCTCTCCCCCC		GTTGCAGATCACGAGGGAAGAGGGGGAAGGGATTCT
ATGCCATAAAAACCGCCTCAACTTAAGCTCTCTCCCCCC		GTTGCAGATCACGAGGGAAGAGGGGGAAGGGATTCT
ATGCCATAAAAACCGCTTCAACTTAAGCTCTCTCCCCCCG	G	TCTAGAAAGACTGGAGTTGCAGATCACGAGGGAAGAGGGGGAAGGGATTCT
ATGCCATAAAAACCGCTTCAACTTAAGCTCTCTCCCCCCG		TCTAGAAAGACTGGAGTTGCAGATCACGAGGGAAGAGGGGGAAGGGATTCT
ATGCCATAAAAACCGCTTCAACTTA	G	TCTAGAAAGACTGGAGTTGCAGATCACGAGGGAAGAGGGGGAAGGGATTCT

MEFs expressing PR65 S401A

Chr-11		Chr-6
ATGCCATAAAAACCGCTTCAACTTAAGCTCTCTCCCCCCGTATCCGGCGAGCC		TCTAGAAAGACTGGAGTTGCAGATCACGAGGGAAGAGGGGGAAGGGATTCT
ATGCCATAAAAACCGCTTCAACTTAAG		TCTAGAAAGACTGGAGCTGCAGATCACGAGGGAAGAGGGGGAAGGGATTCT
ATGCCATAAAAACCGCTTCCACTTAAG		TTTAGAAAGACTGGAGTTGCAGATCACGAGGGAAGAGGGGGAAGGGATTCT
ATGCCATAAAAACCGCTTCAACTTAAGCTCTCTCCCCCCG		TCTAGAAAGACTGGAGTTGCAGATCACGAGGGAAGAGGGGGAAGGGATTCT
ATGCCATAAAAACCGCCTCAACTTAAGCTCTCTCCCCCCG		TCTAGAAAGACTGGAGTTGCAGATCACGAGGGAAGAGGGGGAAGGGATTCT
ATGCCATAAAAACCGCCTCAACTTAAGCTCTCTCCCCCCG		TCTAGAAAGACTGGAGTTGCAGATCACGAGGGAAGAGGGGGAAGGGATTCT
ATGCCATAAAAACCGCTTCAACTTAAGC		TCTAGAAAGACTGGAGTTGCAGATCACGAGGGAAGAGGGGGAAGGGATTCT
ATGCCATAAAAACCGCCTCAACTTAAGCTCTCTCCCCCCG		TCTAGAAAGACTGGAGTTGCAGATCACGAGGGAAGAGGGGGAAGGGATTCT
ATGCCATAAAAACCGCCTCAACTTAAGCTCTCTCCCCCCG		TCTAGAAAGACTGGAGTTGCAGATCACGAGGGAAGAGGGGGAAGGGATTCT
ATGCCATAAAAACCGCTTCAACTTAAGCTCTCTCCCCC		GAGTTGCAGATCACGAGGGAAGAGGGGGAAGGGATTCT
ATGCCATAAAAACCGCTTCAACTTAAGCTCTCTCCCCCCG		TCTAGAAAGACTGGAGTTGCAGATCACGAGGGAAGAGGGGGAAGGGATTCT
ATGCCATAAAAACCGCTTCAACTTAAGCTCTCTCCCCCCG		TCTAGAAAGACTGGAGTTGCAGATCACGAGGGAAGAGGGGGAAGGGATTCT
ATGCCATAAAAACCGCTTCAACTTAAGCTCTCTCCCCCCG		TCTAGAAAGACTGGAGTTGCAGATCACGAGGGAAGAGGGGGAAGGGATTCT
ATGCCATAAAAACCGCCTCAACTTAAGCTCTC		TCTAGAAAGACTGGAGTTGCAGATCACGAGGGAAGAGGGGGAAGGGATTCT
ATGCCATAAAAACCGCCTCAACTTAAGCTCTC		TCTAGAAAGACTGGAGTTGCAGATCACGAGGGAAGAGGGGGAAGGGATTCT
ATGCCATAAAAACCGCCTCAACTTAAGCTCTC		TCTAGAAAGACTGGAGTTGCAGATCACGAGGGAAGAGGGGGAAGGGATTCT
ATGCCATAAAAACCGCCTCAACTTAAGCTCTC		TCTAGAAAGACTGGAGTTGCAGATCACGAGGGAAGAGGGGGAAGGGATTCT
ATGCCATAAAAACCGCTTCAACTTAAGCTCTCTCCCCCC	CGTA	TCTAGAAAGACTGGAGTTGCAGATCACGAGGGAAGAGGGGGAAGGGATTCT
TCTTACAAAAAAAGTCACAAATTAAACCAAAGTATTTTACAGAATTTACTACAAAATGCCATAAAAACCGCTTCA		ACTGGAGTTGCAGATCACGAGGGAAGAGGGGGAAGGGATTCT
ATATTATAAACAATTTCTTACAAAAAAAGTCACAAATTAAACCAAAGTATTTTACAGAATTTACTACAAAATGCCATA		AAAGACTGGAGTTGCAGATCACGAGGGAAGAGGGGGAAGGGATTCT

MEFs expressing PR65 S401D

Chr-11		Chr-6
ATGCCATAAAAACCGCTTCAACTTAAGCTCTCTCCCCCCGTATCCGGCGAGCC		TCTAGAAAGACTGGAGTTGCAGATCACGAGGGAAGAGGGGGAAGGGATTCT
ATGCCATAAAAACCGCCTCAACTTAAGCTCTCTCCCCACACACCAGGTTAGCC	TTTAAGCCTGCCCAGAAGACTCCCGCCCA	TCTAGAAAGACTGGAGTTGCAGATCACGAGGGAAGAGGGGGAAGGGATTCT
ATGCCATAAAAACCGCCTCAACTTAAGCTCTCTCCCCACACACCAGGTTAGCC	TTTAAGCCTGCCCAGAAGACTCCCGCCCA	TCTAGAAAGACTGGAGTTGCAGATCACGAGGGAAGAGGGGGAAGGGATTCT
ATGCCATAAAAACCGCCTCAACTTAAGCTCTCTCCCCACACACCAGGTTAGCC	TTTAAGCCTGCCCAGAAGACTCCCGCCCA	TCTAGAAAGACTGGAGTTGCAGATCACGAGGGAAGAGGGGGAAGGGATTCT
ATGCCATAAAAACCGCTTCAACTTAAGCTCTCTCCCCCC		TCTAGAAAGACTGGAATTGCAGATCACGAGGGAAGAGGGGGAAGGGATTCT
ATGCGATAAAAACCGCTTCAACTTAAGCTCTCTCCCCCC	C	TCTAGAAAGACTGGAGTTGCAGATCACAAGGGAAGAGGGGGAAGGGATTCT
ATGCCATAAAAACCGCTTCAACTTAAGCTCTCTCCCCCC	C	TCTAGAAAGACTGGAGTTGCAGATCACGAGGGAAGAGGGGGAAGGGATTCT
TCTTACAAAAAAAGTCACAAATTAAACCAAAGTATTTTACAGAATTTA		TCTAGAAAGACTGGAGTTGCAGATCACGAGGGAAGAGGGGGAAGGGATTCT
ATGCCATAAAAACCGCTTCAA		CTAGAAAGACTGGAGTTGCAGATCACGAGGGAAGAGGGGGAAGGGATTCT
ATGCCATAAAAACCGCTTCAA		CTAGAAAGACTGGAGTTGCAGATCACGAGGGAAGAGGGGGAAGGGATTCT
ATGCCATAAAAACCGCTTCAACTTAAGCTCTCTCCCCCCG		TCTAGAAAGACTGGAGTTGCAGATCACGAGGGAAGAGGGGGAAGGGATTCT
ATGCCATAAAANCCGCTTCANCTTAAGCTCTCTCCCCCC		AGACTGGAGTTGCAGATCACGAGGGAAGAGGGGGAAGGGATTCT
ATGCCATAAAAACCGCTTCAACTTAAGCTCTCTCCCCCC		CTAGAAAGACTGGAGTTGCAGATCACGAGGGAAGAGGGGGAAGGGATTCT
ATGCGCTAAAAACCGCTTCAACTTTTACTCTCTCCCCCC	CC	GACTGGAGTTACNGACTCGCNGATCGAGGGGGAAGGGGTTCT
ATGCCATAAAAACCGCTTCAACTTAAGCTCTCTCCCCCCG		TCTAGAAAGACTGGAGTTGCAGATCACGAGGGAAGAGGGGGAAGGGATTCT
ATGCCATAAAAACCGCTTCAACTTAAGCTCTCTCCCCCCG		TCTAGAAAGACTGGAGTTGCAGATCACGAGGGAAGAGGGGGAAGGGATTCT
ATGCCATAAAAACCGTTTCAACTTAAGCTCTCTCCCCCC		AGACTGGAGTTGCAGATCACGAGGGAAGAGGGGGAAGGGATTCT
ATGCCATAAAAACCGCCTCAACTTAAGCTC		TTGCAGATCACGAGGGAAGAGGGGGAAGGGATTCT
ATGCCATAAAAACCGCTTCAACTTAAGCTCTCTCCCCCC		AGACTGGAGTTGCAGATCACGAGGGAAGAGGGGGAAGGGATTCT
ATGCCATAAAAACCGCTTCAACTTAAGCTCTCTCCCCCC		AGACTGGAGTTGCAGATCACGAGGGAAGAGGGGGAAGGGATTCT
ATGCCATAAAAACCGCTTCAACTTAAGCTCTCTCCCCCCG		TCTAGAAAGACTGGAGTTGCAGATCACGAGGGAAGAGGGGGAAGGGATTCT
ATGCCATAAAAACCGCTTCAACTTAAGCTCTCTCCCCCCG		TCTAGAAAGACTGGAGTTGCAGATCACGAGGGAAGAGGGGGAAGGGATTCT
ATGCCATAAAAACCGCTTCAA		CTAGAAAGACTGGAGTTGCAGATCACGAGGGAAGAGGGGGAAGGGATTCT
TTCTTACAAAAAAAGTCACAAATTAAACCAAAGTATTTTACAGAATGTACTACAAAATGCCAT		AAAGACTGGAGTTGCAGATCACGAGGGAAGAGGGGGAAGGGATTCT

Supplementary Figure 8.

A

NES : LxxxLxxLxL

1 MAAADGDDSL YPIAVLIDEL RNEDVQLRLN SIKKLSTIAL ALGVERTRSE
51 LLPFLTDTIY DEDEVLLALA EQLGTFTTLV GGPEYVHCLL PPLESLATVE
101 ETVVRDKAVE SLRAISHEHS PSDLEAHFVP LVKRLAGGDW FTSRTSACGI
151 FSVCYPRVSS AVKAELRQYF RNLCSDDTPM VRRAAASKLG EFAKVLELDI
201 VKSEIIPMFS NLASDEQDSV RLLAVEACVN IAQLLPQEDL EALVMPTLRQ
251 AAEDKSWRVR YMVADKFTELQKAVGPEITKTDLVPAFQNL MKDCEAEVRA
301 AASHKVKEFC ENLSADCREN VIMTQILPCI KELVSDANQH VKSALASVIM
351 GLSPILGKDNTIEH**LLPLFLAQL**KDECPEV RLNIISNLDC VNEVIGIRQL
401 SQSLLPAIVE LAEDAKWRVR LAIIEYMPLL AGQLGVEFFD EKLNSLCMAW
451 LVDHVYAIRE AATSNLKKLV EKFGKEWAHA TIIPKVLAMS GDPNYLHRMT
501 TLFCINVLSE VCGQDITTKH MLPTVLRMAG DPVANVRFNV AKSLQKIGPI
551 LDNSTLQSEV KPILEKLTQD QDVDVKYFAQ EALTVLSLA

B

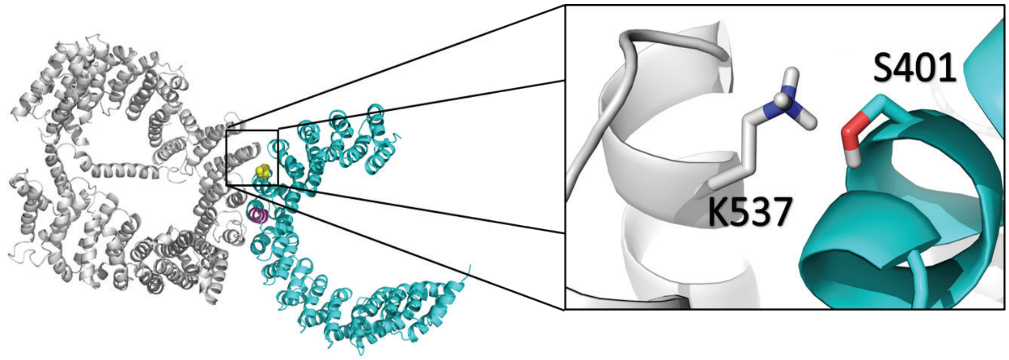

C

PR65: - L_{365}LPLFLAQL_{373} -

NES consensus: - L-X-X-LF-XX-LSV -

A: Structural (15 HEAT repeats)
B: Regulatory
C: Catalytic subunit

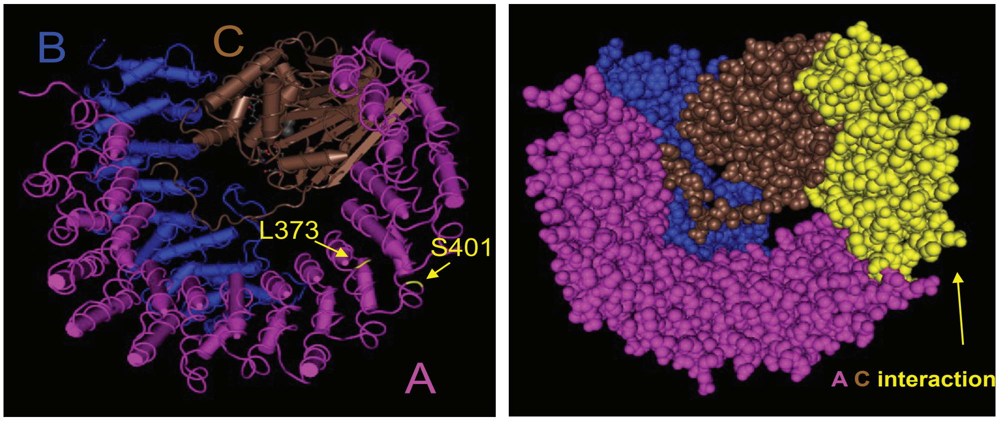

Supplemental Figure 9.

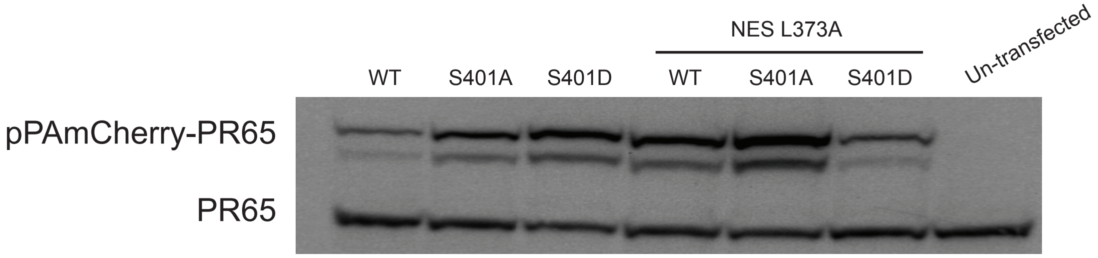

Supplementary Figure 10.

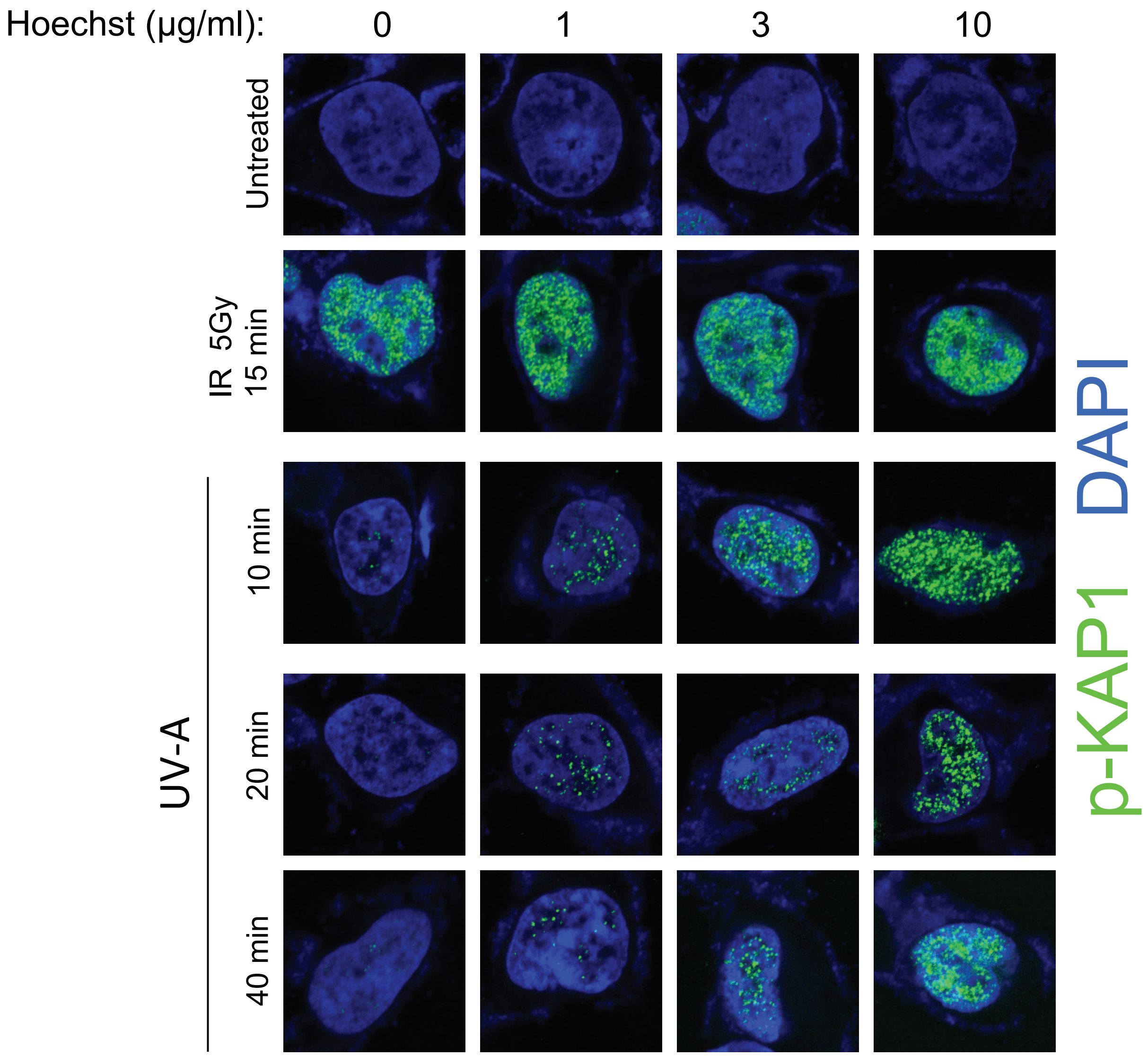

Supplementary Figure 11.

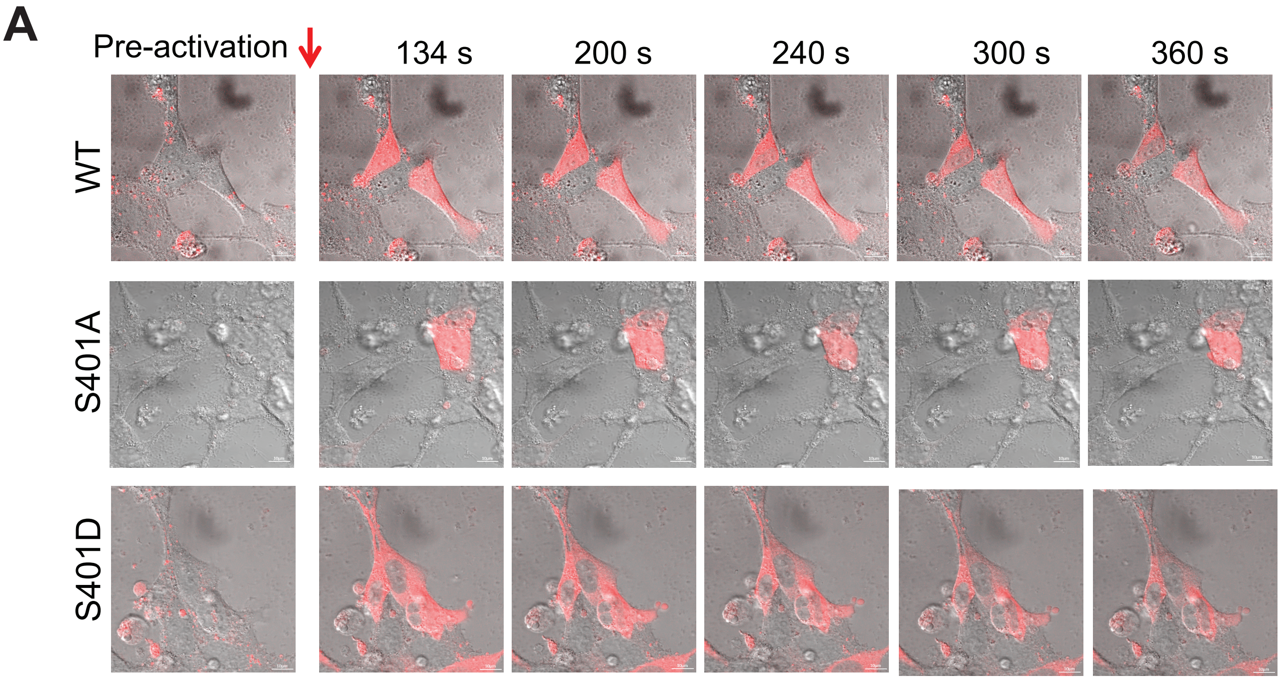

Supplementary Figure 12.

A

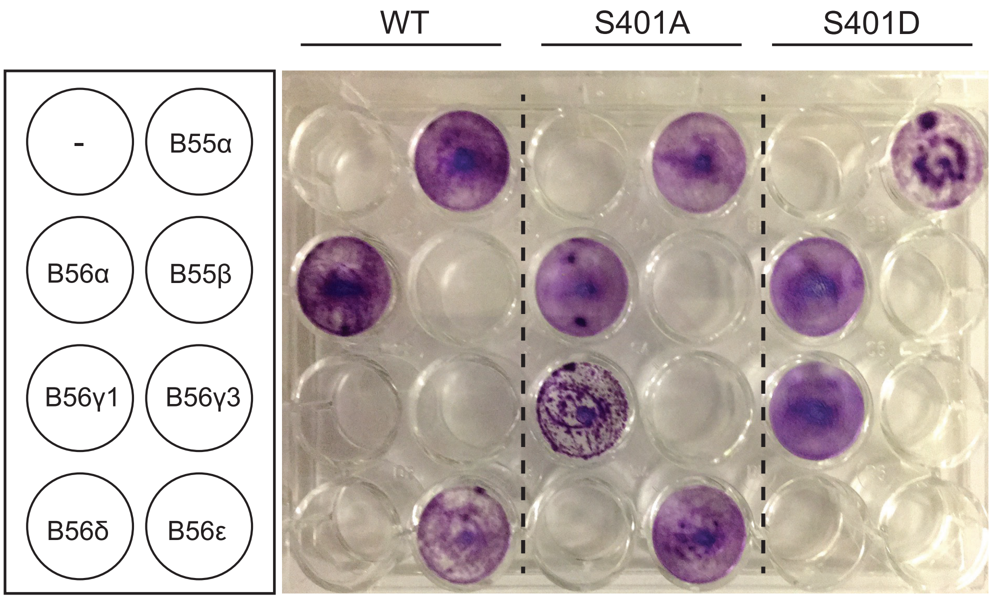

B

B-subunit	WT	S401A	S401D
B55α/PPP2R2A	+	+	+
B56α/PPP2R5A	+	+	+
B56β/PPP2R5B	-	-	-
B56γ1/PPP2R5C	-	+	+
B56γ3/	-	-	-
B56δ/PPP2R5D	-	-	-
B56ε/PPP2R5E	+	+	-

Supplementary Table 1.

WT

Row	gaussian1	gaussian2	gaussian3
mu	112.6713334	47.99697261	172.1115854
mu_CI_lower	112.1608046	47.03594875	159.9828084
mu_CI_upper	113.1693958	48.97239987	186.0252576
sigma	20.57601541	13.99117091	94.11453542
sigma_CI_lower	20.13991044	13.10672902	85.39626477
sigma_CI_upper	21.02688002	14.89745807	102.5372019
proportion	0.8072826	0.151702945	0.041014455
proportion_CI_lower	0.796783762	0.142667002	0.034361264
proportion_CI_upper	0.817558008	0.16064956	0.047994046

S401A

Row	gaussian1	gaussian2	gaussian3	gaussian4	gaussian5
mu	107.8401084	61.9504846	262.3366658	142.5177	48.63107
mu_CI_lower	106.6717451	60.16653504	203.624445	131.1313	44.22629
mu_CI_upper	115.4548081	68.23947331	377.3792976	165.2139	107.7963
sigma	21.32404405	9.207093964	124.878936	42.67979	21.57652
sigma_CI_lower	18.25635918	7.568911911	80.64580349	32.13473	8.859971
sigma_CI_upper	23.07417712	24.27469864	170.3866235	51.42518	43.28659
proportion	0.569086021	0.115927342	0.007074914	0.198092	0.109819
proportion_CI_lower	0.357837158	0.080946403	0.002256971	0.10822	0.042099
proportion_CI_upper	0.624558375	0.346681673	0.017940919	0.271828	0.196641

S401D

Row	gaussian1	gaussian2	gaussian3
mu	106.0315687	46.76675558	174.8895841
mu_CI_lower	105.4597517	46.39652219	165.5553866
mu_CI_upper	106.6041131	47.13127314	185.3803763
sigma	20.54104028	9.570053569	93.10054925
sigma_CI_lower	19.98778326	9.266241556	87.48585699
sigma_CI_upper	21.09092988	9.868076039	98.51755827
proportion	0.607900591	0.335846752	0.056252658
proportion_CI_lower	0.597047248	0.326093943	0.049496932
proportion_CI_upper	0.619078528	0.345248898	0.063379692

Syn

Row	gaussian1	gaussian2	gaussian3
mu	119.0488805	44.52886136	171.5183648
mu_CI_lower	118.570992	41.67076681	161.6553723
mu_CI_upper	119.597509	47.40926267	235.400503
sigma	18.32874438	20.95980558	91.48017913
sigma_CI_lower	17.97499435	18.70377407	27.44910405
sigma_CI_upper	18.77843553	23.45894998	129.842548
proportion	0.925191784	0.055257503	0.019550713
proportion_CI_lower	0.91788522	0.049143018	0.007864581
proportion_CI_upper	0.937139109	0.064220123	0.02474155

SupplementaryTable 2.

One-way ANOVA results

Table analysed	WT, S401A and S401D	NES, NES-S401A and NES S401D
Repeated measures ANOVA summary		
Assume sphericity?	No	No
F	12255	159153
P value	<0.0001	<0.0001
P value summary	****	****
Statistically significant (P < 0.05)?	Yes	Yes
Geisser-Greenhouse's epsilon	0.5335	0.5574
R squared	0.9227	0.9936

Sidak's multiple comparisons test	Mean Diff.	95.00% CI of diff.	Significant?	Summary	Adjusted P Value
WT vs. S401A	0.007376	0.007125 to 0.007627	Yes	****	<0.0001
WT vs. S401D	0.02003	0.01963 to 0.02043	Yes	****	<0.0001
NES vs. S401A-NES	0.06693	0.06650 to 0.06736	Yes	****	<0.0001
NES vs. S401D-NES	0.07151	0.07121 to 0.07180	Yes	****	<0.0001

Test details	Mean 1	Mean 2	Mean Diff.	SE of diff.	n1	n2	t	DF
WT vs. S401A	0.03816	0.03079	0.007376	0.000112	1027	1027	65.83	1026
WT vs. S401D	0.03816	0.01813	0.02003	0.0001787	1027	1027	112.1	1026
NES vs. S401A-NES	0.09468	0.02774	0.06693	0.000192	1027	1027	348.7	1026
NES vs. S401D-NES	0.09468	0.02317	0.07151	0.000132	1027	1027	541.6	1026

Sinple Linear regression analysis

	WT	S401A	S401D	NES	S401A-NES	S401D-NES
Slope	-0.00006907	-0.00002349	0.000007893	-0.0000744	-0.00001349	-0.00004356
Y-intercept	0.04652	0.03363	0.01717	0.1037	0.02937	0.02844
X-intercept	673.5	1431	-2176	1394	2178	652.9
1/slope	-14478	-42564	126702	-13441	-74145	-22958
Std. Error						
Slope	8.245E-07	0.000000587	3.543E-07	0.000001373	6.394E-07	6.069E-07
Y-intercept	0.0001153	0.00008206	0.00004952	0.000192	0.00008938	0.00008484
95% Confidence Intervals						
Slope	-7.068e-005 to -6.745e-005	-2.464e-005 to -2.234e-005	7.198e-006 to 8.587e-006	-7.709e-005 to -7.171e-005	-1.474e-005 to -1.223e-005	-4.475e-005 to -4.237e-005
Y-intercept	0.04630 to 0.04675	0.03347 to 0.03379	0.01708 to 0.01727	0.1033 to 0.1041	0.02920 to 0.02955	0.02827 to 0.02860
X-intercept	660.8 to 686.9	1370 to 1499	-2398 to -1990	1349 to 1441	2003 to 2389	638.6 to 667.9
P value	<0.0001	<0.0001	<0.0001	<0.0001	<0.0001	<0.0001
