# Supplementary material for: ATM phosphorylates PP2A subunit A resulting in nuclear export and spatiotemporal regulation of the DNA damage response
Source: Cell Mol Life Sci. 2022 Nov 24;79(12):603. doi: 10.1007/s00018-022-04550-5 (PMC9700600; doi:10.1007/s00018-022-04550-5)
Supplement: Supplementary file 17 — Supplementary file17 (PDF 190 KB) [file 18_2022_4550_MOESM17_ESM.pdf]

Supplementary Information

ATM phosphorylates PP2A subunit A resulting in nuclear export and spatiotemporal regulation of the DNA damage response

Amrita Sule[1,2], Sarah E. Golding[1], Syed F. Ahmad[1,2], James Watson[1], Mostafa H. Ahmed[3], Glen E. Kellogg[3,7], Tytus Bernas[4], Sean Koebley[5], Jason C. Reed[5,7], Lawrence F. Povirk[6,7], and Kristoffer Valerie[1,2,7,*]

Departments of [1]Radiation Oncology, [2]Biochemistry and Molecular Biology, [3]Medicinal Chemistry, [4]Anatomy, [5]Physics, [6]Pharmacology and Toxicology,
and the [7]Massey Cancer Center,
Virginia Commonwealth University, Richmond, VA 23298, USA

Materials and Methods:

Plasmids:

Plasmids	Source	Provided by
pMIG-Aalpha	Addgene #10884	William Hahn
Flag-hCRM1	Addgene #17647	Xin Wang
pmCherry-C1-RanQ69L	Addgene #30309	Jay Brenman
pCSCMV:tdTomato	Addgene #30530	Gerhart Ryffel
Human TERT (LOX-TERT-iresTK	Addgene #12245	Didier Trono
pcDNA3-mRuby2	Addgene #40260	Michael Lin
pGEX-2T	GE Life Sciences #28954653	
YFP-ATM	Reference (1)	David Chen
pBabe-hygroPyMT	Addgene #22305	Filippo Giancotti
pPAmCherry-a-tubulin	Addgene #31930	Vladislav Verkhusha
LentiCRISPR v2	Addgene plasmid # 52961	Feng Zhang
LentiCRISPRv2 hygro	Addgene plasmid # 98291	Brett Stringer
pCEP-4HA-B56 alpha	Addgene #14532	David Virshup
pCEP-4HA-B56 gamma3	Addgene #14535	David Virshup
pCEP-4HA-B56 gamma1	Addgene #14534	David Virshup
pCEP-4HA-B56 epsilon	Addgene #14537	David Virshup
pCEP-4HA-B56 delta	Addgene #14536	David Virshup
pCEP-4HA-B56 beta	Addgene #14533	David Virshup
pCEP-4HA-B55 alpha	Created by replacing B56 alpha from pCEP-4HA-B56 beta with B55 alpha. The B55 alpha cDNA was optained from pUC19-hPPP2R2A by PCR.	This study

Antibodies and Reagents:

Antibodies and Reagents	Company	Catalog no.
anti-ATM	Cell Signaling Technology	2873
anti-AKT	Cell Signaling Technology	2920
anti-p(S473)-AKT	Cell Signaling Technology	9271
anti-PP2A-C	Cell Signaling Technology	2038
anti-pS1390-gH2AX	Cell Signaling Technology	9718
anti-GST	Cell Signaling Technology	2624
anti-γ-H2AX	Millipore Sigma	05-636
anti-GAPDH	Millipore Sigma	MAB374
anti-Flag	Millipore Sigma	F1365
anti-53BP1	Novus Biologicals	NP100-304
anti-Rad51	Novus Biologicals	NB100-148
anti-PyMT	Novus Biologicals	NB100274955
anti- p(S4/S8)-RPA	Bethyl laboratories	A300-245A
anti-ERK2	Santa Cruz Biotechnology	sc-154
anti-pERK1/2	Santa Cruz Biotechnology	sc-7383
anti-CRM1	Santa Cruz Biotechnology	sc-74454
Alexa Fluor 594 goat anti-mouseIgG	Life Technologies	R37121
Alexa Fluor 680 goat anti-rabbit IgG	Life Technologies	A-21076
Alexa Fluor 680 goat anti-mouse IgG	Life Technologies	A-21057
Dylight 800 anti-mouse IgG	Cell Signaling Technology	5257
Dylight 800 anti-rabbit IgG	Cell Signaling Technology	5151
Leptomycin B	Millipore Sigma	L2913
Camptothecin	Millipore Sigma	7689-03-4
KU-60019	Selleckchem	S1570

Supplementary Methods and Data

DNA repair assays. pEGFP-C1 (Clontech) plasmid was digested with BamHI and BglII to generate a 51-bp fragment which was inserted into the BamHI site of pCSCMV:tdTomato to generate a SmaI restriction site immediately downstream from the BamHI site. The modified pCSCMV:tdTomato + linker plasmid was linearized with SmaI followed by BamHI to create incompatible DNA ends and gel purified prior to use.

pCSCMV:tdTomato+linker plasmid (1 μg) was transfected into the MEFs (from a 80% confluent 6-cm plate) and cells divided equally into the wells of a 12-well plate. Cells were collected at 2, 4 and 8 hours from the media and pooled with the remaining cells after trypsinization. A small fraction was used to determine RFP+ cell numbers using a Nexcelom cellometer (Nexcelom Bioscience). Remaining cell pellets were re-suspended in 15% sucrose, 50 mM Tris-HCl, 25 mM EDTA buffer supplemented with 0.1% TritonX-100 and treated with RNase. Cell lysates were incubated at 50°C for 3-4 hours. Equal amount of re-suspension buffer was combined with equal volume of 0.1% SDS and 50 μg/ml of Proteinase K. Samples were incubated at 60°C overnight. This was followed by phenol chloroform treatment and DNA was precipitated with 1:1 isopropanol. The level of repaired DNA was assessed by quantitative real-time PCR, using an ABI 7900HT (Applied Biosystems) and SYBR green detection. The (Forward 5' CGGAGCAAGCTTGATTTAGGTG 3' and Reverse 5' CGCATGAACTCTTTGATGACCTC 3') primers were used to analyze the repair junction between BamHI and SmaI with primers for ampicillin gene (Forward 5'TGTGCAAAAAAGCGGTTAGCT 3' and Reverse 5'GCGGCCAACTTACTTCTGACA 3') were used for template normalization.

In vitro end joining assay was carried out at 37°C in 50 mM triethanolammonium-acetate pH 7.5, 1 mM ATP, 1 mM dithiothreitol, 50 µg/ml BSA, 1.3 mM magnesium acetate and dNTPs at 100 µM each. Typically, a 16-µl reaction contained 10 µl of whole-cell extract, resulting in a final concentration of 8 mg/ml protein, 66 mM potassium acetate and 16% glycerol, and an effective Mg^{2+} concentration of 1 mM. The substrate was an internally labeled plasmid with partially cohesive ends, prepared as

described (2,3). Homologous recombination GFP assay (DR-GFP) was carried out as previously described by determining GFP+ cells by flow cytometry (4,5). PR65$^{flox/flox}$ MEFs were stably transfected with linearized DR-GFP plasmid and selection with puromycin followed by infection with WT, S401A or S401D pMIG-Aα (PR65) lentivirus. Pooled GFP+ cells were then infected with Ad-CMV-Cre to remove the endogenous PR65 (see Fig. 1).

CRISPR-Cas9 DSB repair and chromosomal translocation assay was performed as described (6,7). PR65-KO MEFs (WT, S401A and S401D) were infected with lentivirus expressing Cas9 and gRNA (Rosa26) and (Rosa26; H3f3b), respectively, by introducing one or two guide RNAs (5′-GTTGGCTCGCCGGATACGGG-3′ for H3f3b; 5′-ACTCCAGTCTTTCTAGAAGA-3′ for Rosa26) into LentiCRISPR v2 (Addgene plasmid #52961) and LentiCRISPRv2 hygro (Addgene Plasmid #98291). Plasmids were sequenced to confirm the correct gRNA insertions. To generate mixes of stable cell populations lentivirus was made in HEK293T cells and MEFs infected followed by selection with puromycin or hygromycin as described (8,9). The Rosa26 and H3f3b primers used for PCR were those described (7). Amplified products were cloned in pGEM®-T Easy (Promega) and sequenced to verify translocation and junction sequences.

Chromosome translocation and amplicon length heterogeneity were quantified using high-speed atomic microscopy (HSAFM) with sample preparation and execution of the technique described in detail previously (10-12). Briefly, a tip with a <10 nm sharpened point was rastered across a surface, and the height of the tip measured to yield a three-dimensional map of the sample features with nanoscale resolution (13). Our high-speed

atomic force microscope (Bristol Nanodynamics) routinely acquires 2 μm x 2 μm images with single-nanometer resolution at a rate of 1 image per second (14). To deposit DNA onto an atomically flat surface for imaging, we first purified the DNA from PCR reactions using Ampure (Beckman Coulter), then added $MgCl_2$ to facilitate adhesion of DNA to negatively-charged mica. The resulting DNA solution was drop-deposited onto freshly cleaved mica (Ted Pella, Inc), dried with compressed air, and baked for 10 minutes at 120°C. Following HSAFM imaging, we employed a custom line-by-line processing program to flatten the images, which is required to account for mechanical and thermal vibration. To quantify DNA lengths observed in the flattened images, we used custom computer vision programming to automate DNA tracing and filter out strands that did not meet certain quality metrics, e.g. strands that were branched or circular. Analysis of traced length distributions was performed by fitting Gaussian mixture models, then analyzing the Bayesian information criteria to determine the number of Gaussian distributions that produced the best fit. The 95% confidence interval for the mean of each fitted Gaussian was determined using a bootstrapping approach with n = 10,000. All programming and analyses were conducted using MATLAB.

Immunoprecipitation. Flag-PR65 was immunoprecipitated from HEK293 cells stably expressing pMIG-Aalpha WT, S401A or S401D with anti-Flag M2 beads (Sigma Aldrich, Cat #F2426) in lysis buffer with Halt™ protease and phosphatase inhibitor cocktail (ThermoFisher Scientific, Cat #78440). YFP-ATM was immunoprecipitated by using the GFP-Trap Magnetic Agarose beads (Chromotek, Cat #gtma-10).

***In vitro* kinase assay.** YFP-ATM was immunoprecipitated from stably transfected HEK293 cells by GFP-TRAP (Chromotek). The immunoprecipitates were suspended in kinase assay buffer containing 2 μCi of [γ-^{32}P] ATP, 20 μM unlabeled ATP, and GST-substrates (GST-PR65 or GST-p53). GST-PR65 has GST fused to full-length PR65 and GST-p53 expresses the first 100 amino acids of p53 (15). GST-fusions were purified from extracts of BL21 (DE3)/pLysS cells using Glutathione Sepharose 4B beads as recommended by the manufacturer (GE Healthcare Life Sciences). The kinase reaction was conducted at 30°C for 20 minutes and stopped by adding 2X Laemmli loading buffer. Samples were separated on 10% polyacrylamide gels, followed by drying of the gel onto 3M paper, exposed to a screen, scanned by a Phosphorimager (Typhoon) and the signal quantified (15).

Microscopy and cell imaging. For DNA repair foci assay, cells were grown on Lab-Tek (Naperville, IL) glass slides. After treatment, cells were fixed with 3% paraformaldehyde, made permeable with 0.5% Triton-X-100 in phosphate buffered saline (PBS) and blocked with casein (ThermoFisher Scientific Cat #37528). Cells were stained with primary antibodies diluted in casein blocker followed by secondary antibodies, and nuclei were counterstained with DAPI (1 μg/ml) and mounted in Vectashield® mounting medium (Vector Labs). Cells were imaged and analyzed using Zeiss LSM 710 imaging system in the VCU Microscopy Facility.

For photoactivation studies to capture representative images, HEK293 cells were transfected with pPAmCherry-PR65-WT, -S401A and -S401D in glass bottom dishes and 48 h post-transfection sub-nuclear ROI (4 μm x 4 μm), marked by the squares,

were photoactivated by 405 nm laser (242.8 μJ) and followed over time (<300 s) by live cell imaging using a Zeiss LSM 710 microscope with images acquired at 63x. For quantitative photoactivation studies, HEK293 cells, stably transfected with linearized pPAmCherry-PR65 (WT, S401A and S401D, NES, S401A-NES and S401D-NES), were cultured under G418 (500 μg/ml) selection in a glass bottom dish for 48 h. Next, the cells were imaged with Zeiss LSM 880 confocal system, based on Axio Z1 inverted stage and equipped with 40x PlanApo oil immersion objective (NA = 1.4), 405 nm diode laser (30 mW) and 561 nm DPSS laser (20 mW). Fluorescence of mCherry was excited with the latter laser attenuated to 0.8% and detected in 570 – 620 nm range with a multi-anode (spectral) GaAsP hybrid detector in the integrative mode at 950V gain. Time series of transmitted light and fluorescence images were collected with 0.28 μm pixel size, 3.07 μs dwell time (236 μs frame time) and 256x256 pixel frame size. Following first 25 frames 2.8x2.8 μm regions in cell nuclei were irradiated with 325.2 μJ of 405 nm light. Next, imaging mCherry fluorescence resumed for 277 s (1175 frames). Up to 7 irradiation regions were selected in each field of view using transmitted light image as the reference.

Temporal averages of the series were used for initial segmentation of cytoplasmic regions where mCherry fluorescence was stronger than in the corresponding irradiated nuclei. Segmentation thresholds were calculated by adding average and standard deviation (multiplied by 0.3) of fluorescence in the irradiated nuclear regions. The resulting binary masks were inverted and used to limit conditional dilation of the seeds (10x10 pixels) corresponding to the irradiated regions. Next, the initial nuclear masks created in this manner, were used to construct 3-pixel wide regions around them, at the

distance of 4 pixels. Products of these regions and the result of the initial cytoplasm segmentation were used as cytoplasmic counterparts of the nuclear masks. These initial masks were updated tor every image in the post-irradiation series by conditional binary closing (2 iterations) of nuclear masks and according to construction of the cytoplasmic masks. Median intensity of the pre-irradiation images was used as background estimator. Average nuclear and cytoplasmic mCherry fluorescence intensities were corrected for background. The respective nuclear/cytoplasmic (N/C) ratios were averaged over cell populations.

Proximity Ligation assay. Proximity ligation assay was performed as per manufacture's recommendations (Duolink® using PLA® technology from Sigma Aldrich). PR65 KO MEFs expressing Flag-tagged WT PR65 were seeded on Lab-Tek (Naperville, IL) glass slides and treated with Leptomycin B (2 ng/ml) or not for 3 hours, irradiated with 10 Gy or not, or left untreated. Cells were fixed after 15 min followed by rabbit anti-PR65 and mouse anti-CRM1 antibodies to determine the extent of the PR65-CRM1 interaction. Images were obtained by confocal microscopy.

Supplementary Figure Legends

Supplementary Figure S1. SV40 small-t and polyoma Middle-T inhibit PP2A and blocks radiation-induced AKT activation. (**A**) SV40 t (*SVST*) and Polyoma MT (*PyMT*) antigens are known to replace the PP2A-B subunit and bind to the PP2A-A and C Core enzyme to inhibit PP2A activity and increase AKT phosphorylation (16) (**B**) Inhibition of ATM does not reduce pAKT (S473) levels in HEK293T cells but does in U87, U1242, and HEK293 cells. U87, U1242, HEK293, and 293T cells were treated with or without KU-60019 (3 μM) for 1 hour. Whole cell extracts were separated on an SDS-PAGE gel and analyzed by western blotting using anti-pAKT (S473) and anti-AKT antibodies. (**C**) U1242 and (**D**) U87 cells expressing PyMT antigen show diminished ATM-mediated inhibition of pAKT (S473) levels after radiation. U1242 and U87 cells were infected with pBabe-hygroPyMT vector or control vector pBabe-hygro. Infected cells were pre-treated with or without KU-60019 (3 μM) for 1 hour followed by 5 Gy and whole cell extracts prepared at one-hour post-IR were separated on an SDS-PAGE gel and analyzed by western blotting using anti-p-AKT and anti-AKT antibodies. (**E**) U1242 and U87 cells infected with PyMT virus are positive for virus integration. Chromosomal DNA was used to as template for PCR to show integration of PyMT sequences normalized with β-actin primers. (**F**) Treatment of nuclear extract with okadaic acid increases pAKT levels. Nuclear extract was prepared from human glioma U87 cells and incubated with OA (3, 10, 30, or 100 nM) or not for 2 hours. Samples were then separated by SDS-PAGE and transferred to a membrane followed by sequential western blotting with anti-pAKT (S473) and AKT antibodies. Quantification of band intensity was done by densitometric scanning.

Supplementary Figure S2. ATM kinase phosphorylates PR65 in vitro. (**A**) HEK293 cells were transfected with YFP-ATM +/- mRuby2-PR65 constructs and sorted with only YFP-ATM/PR65 (S401A) cells surviving suggesting that ATM and PR65 S401A, but not WT or D, can be stably co-expressed in HEK293 cells whereas S401 phosphorylation or expression of S401D phosphor-mimetic is not tolerated with over-expressed ATM. FT; flow-through. (**B**) Whole cell extracts of HEK293 cells expressing YFP-ATM were immunoprecipitated with GFP-TRAP (ChromoTek). (**C**) In vitro kinase assay was performed by suspending YFP-ATM immunoprecipitate w/o co-expressed PR65 in buffer with [γ-^{32}P] ATP, 20 μM unlabeled ATP, GST-substrates (GST-$PR65_{FL}$ or GST-$p53_{100}$) with or without KU-60019 (1 μM) for 1 hour. Samples were separated on an SDS-PAGE gel and then analyzed by a Typhoon phosphoimager (PerkinElmer) (*top panel*). Phosphorylation of p53 and PR65 is reduced in the presence of KU-60019 (lane 3 and lane 6) relative to controls (lane 2 and 5), respectively. Coomassie Blue (*CB*) - stained image of the gel showing GST-p53 and GST-PR65 proteins (*bottom panel*).

Supplementary Figure S3. Growth and irradiation signaling is impaired in PR65 S401 mutant cells. (**A**) AKT phosphorylation is increased in S401D cells after insulin stimulation. MEFs (WT, S401A and S401D) were serum starved for 16 hours and then treated with insulin for 15-, 30- or 60-min. Whole cell extracts were separated on an SDS-PAGE gel and analyzed by western blotting with anti-pAKT (S473), anti-AKT, anti-GAPDH (loading control) antibodies (*left panel*). Western blots were quantified using Image Studio Lite. p-AKT (S473) levels were normalized to total AKT protein levels (*right panel*). (**B**) S401D cells have increased growth rate relative to S401A and WT.

MEFs were grown at different dilutions in 96-well plates. Cells were analyzed by Cell titer-Glo® at 2, 5, and 7 days to determine growth. Data points; Growth, Relative Luminescence Units (RLU). Error bars; mean ± SEM (n = 3). Statistical analysis was carried out using unpaired, two-tailed *t*-tests. *P*-values expressed as *($P < 0.05$) and **($p < 0.01$) were considered significant. At 7 days, WT vs. S401D; p=0.0026, S401A vs. S401D; p= 0.0118. (**C**) ERK phosphorylation is elevated in S401D cells. Whole cell extracts of MEFs were separated on an SDS-PAGE gel and analyzed by western blotting using anti-pAKT, anti-AKT, anti-pERK and anti-ERK antibodies. pAKT (S473) levels were normalized to total AKT protein levels and anti-pERK (T202/Y204) levels were normalized to total ERK levels. Increased pERK levels correlate with increased growth of S401D cells. (**D**) S401 cells show dampened response to irradiation. MEFs were exposed to 2 Gy of ionizing radiation and collected after 5, 15, 30 and 60 minutes. Whole cell extracts were separated on an SDS-PAGE gel and analyzed by western blotting using anti-pAKT, anti-AKT, anti-γ-H2AX, and anti-GAPDH antibodies (*top panel*). Western blots were quantified using Image Studio Lite (Li-Cor). p-AKT levels were normalized to total AKT protein levels and γ-H2AX levels were normalized to GAPDH (*bottom panels*).

Supplementary Figure S4. S401 mutant cells show impaired replication restart. (**A**) S401D cells quickly exit mitosis after mitotic synchronization. Cells were arrested in M phase with 16 hours of nocodazole treatment. Mitotic cells were collected by shake-off, reseeded in complete media, and collected after 1, 2 and 4 hours for western blotting with anti-pPLK1 (T210) antibody, a marker of mitotic entry. (**B and C**) Faster pPLK1 dephosphorylation occurs in S401D cells expected to speed up mitotic exit.

Altered PP2A-S401D target specificity/activity might explain quicker exit from mitosis. (**D**) S401D cells show delayed replication restart. MEFs were treated with 1 mM of hydroxyurea (HU) for 2 hours followed by drug wash-out and collected at 0, 1, and 6 hours after wash-out. Western blotting with antibodies specific for pRPA (S4/S8) and γ-H2AX normalized to total GAPDH.

Supplementary Figure S5. S401 mutant cells are more sensitive to camptothecin than wild type MEFs. (**A**) WT, S401A and S401D MEFs were treated with CPT and analyzed by Cell titer-Glo® assay at 96 hours to determine cell survival. Error bars; mean ± SEM (n = 3). CPT IC_{50} is presented in the table.

Supplementary Figure S6. PCR and western blot analyses to verify floxing of endogenous PR65 sequences in MEFs stably transfected with DR-GFP. PCR screening with primers P1 and P3 generated a 263-bp product for the WT allele and a 417-bp product for the CKO allele (see Legend to Fig. 1A). In cells where Cre was expressed, primer pair P3 and P2 generated a 587-bp product for the PR65 KO allele (*top panel*). Whole cell extracts of WT, S401A, and S401D MEFs with integrated DR-GFP infected with Ad-Cre or not were analyzed by western blotting with anti-PR65 antibody (*bottom panel*). Notice that the bottom PR65 band is missing after floxing indicating the loss of the endogenous allele. In addition, Flag-PR65 levels in WT, S401A, and S401 are very similar (compare with **Fig. 1E**).

Supplementary Fig S7. Translocation between DSBs (CRISPR-Cas9) at Rosa26 (Chr 6) and H3f3b (Chr 11). Sequences of Chr(11) breakpoint junction from WT (**A**), S401A (**B**) and S401D (**C**) cells. Reference sequence is highlighted at the top (*blue*). The remaining DNA sequences represent individual translocations recovered by PCR and subject to Sanger sequencing. Nucleotide insertions are marked in red. Nucleotide deletions are represented as gaps. Micro-homology is denoted by cyan highlighted nucleotides.

Supplementary Figure S8. Putative PR65 NES in close proximity to S401. (**A**) PR65 has a putative nuclear export sequence (highlighted in red) identified using the online tool NetNES1.1 (http://www.cbs.dtu.dk/services/NetNES/). (**B**) Model for PR65-CRM1 interaction via PR65-S401 and CRM1-K537. PR65-CRM1 co-crystal modeling showing a possible key interaction between PR65-S401 and CRM1- K537 expected to increase after S401 phosphorylation. PR65-CRM1 is colored in cyan whereas CRM1 is colored in silver. (C) PR65 S401 phosphorylation might expose a buried PR65 NES. A putative NES is located in the inter-repeat loop between HEAT domains 10-11 of PR65 and in close proximity to S401. Co-crystal structure of PP2A with PR65 structural (A), catalytic (C), and (B) regulatory subunits with S401 and putative NES at L373 shown in yellow (*left panel*). Interaction domains between PR65 (A) and catalytic (C) subunits (*right panel*). The *yellow* portion of PR65 marks the domain directly interacting with the PP2A catalytic subunit (*brown*). PR65-S401 is located at the rim whereas L373 is buried deeper at the interface between the PR65 and the PP2A catalytic subunit (*left panel*).

Supplementary Figure S9. Expression of PR65-NES and S401A/NES and S401D/NES mutants. Transfection of HEK293 followed by western blotting of extracts shows approximately similar expression levels with anti-Flag and -PR65 antibodies. Notice the size difference between endogenous PR65 and much larger PAmCherry-PR65 proteins.

Supplementary Figure S10. Hoechst 33258 pretreatment sensitizes cells to DNA damage. HEK293 cells were exposed to IR (5 Gy, 15 minutes) with or without Hoechst 33258 (1, 3, or 10 μg/ml; 3 hours) treatment or treated with UV-A (0.20 J/m^2/s for 10, 20 or 40 minutes) with or without Hoechst 33258 (1, 3 or 10 μg/ml; 3 hours) treatment or treated with Hoechst 33258 alone (1, 3 or 10 μg/ml; 3 hours). Cells were immunostained with anti-pKAP1 (S824) and counterstained with DAPI. Images were acquired at 63x power.

Supplementary Figure S11. PR65 nuclear - cytoplasmic shuttling. Nuclear and cytoplasmic fluorescence over time. HEK293 cells were transfected with pPAmCherry-PR65 (WT, S401A and S401D) in a glass bottom dish. Forty-eight hours post-transfection sub-nuclear ROI (4 μm x 4 μm) were photo-activated by 405 nm laser and cells monitored over time by live cell imaging performed on Zeiss LSM 710 and images were acquired at 63x.

Supplementary Figure S12. Tolerability of PR65 B subunit overexpression in PR65 MEFs. PR65-WT, -S401A, and -S401D MEFs were transfected with pCEP-4HA-

B56 alpha, -B56 gamma3, -B56 gamma1, -B56 epsilon, -B56 delta, -B56 beta, or -B55 beta and selected for hygromycin (200 μg/ml) resistance. pCEP plasmids are maintained episomally as EBVori-EBNA plasmids in transfected MEFs (17). After several weeks of serial passaging under selection cells were fixed and stained with Crystal Violet (**A**). Results are summarized in (**B**).

Supplementary Table S1. Statistics for AFM studies (Figure 5C). Four samples were amplified: wild-type (WT, *n* = 11,303 measured strands), S401A (*n* = 6,530), S401D (*n* = 12,160), and a synthetic fragment (Syn, *n* = 6,035) of equivalent length and sequence as WT. Gaussian distribution of 3 populations for WT, S01D and Syn and five populations for S401A was analyzed. Analysis of traced length distributions was performed by fitting Gaussian mixture models then analyzing the Bayesian information criteria to determine the number of Gaussian distributions that produced the best fit. The 95% confidence interval for the mean of each fitted Gaussian was determined using a bootstrapping approach with *n* = 10,000.

Supplementary Table S2. Statistics for photoactivation (Figure 8C). One-way ANOVA test was conducted for the nuclear to cytoplasmic intensity ratios in the time series studies for WT, S401A and S40D as well as NES, NES-S401A and NES- S401D groups. On Multiple comparison test, the differences between WT vs S401A, WT vs S401D, NES vs NES-S401A and NES vs NES-S401D were highly significant. We also performed simple linear regression on the time series of the N/C intensities and found that the slopes of WT, S401A and S401d as well as NES, NES-S401A and NES - S401D were significant different from each other. The mean nuclear to cytoplasmic intensity ratios, and standard deviation were tabulated for the time series photoactivation experiment. The time series is in seconds before and after illumination (photoactivation). The sample size was as n=28 for WT, n=42 for S401A, n=22 for S401D, n=11 for WT-NES, n=23 for S401-NES and n=10 for S401D-NES.

Supplementary videos. PAmCherry-PR65-WT, -S401A, and -S401D transfected HEK293. Videos of representative photoactivated WT (video 1), S401A (video 2), and S401D (video 3) cells undergoing shuttling.

References

1. So S, Davis AJ, Chen DJ. Autophosphorylation at serine 1981 stabilizes ATM at DNA damage sites. J Cell Biol **2009**;187:977-90
2. Povirk LF, Zhou RZ, Ramsden DA, Lees-Miller SP, Valerie K. Phosphorylation in the serine/threonine 2609-2647 cluster promotes but is not essential for DNA-dependent protein kinase-mediated nonhomologous end joining in human whole-cell extracts. Nucleic Acids Res **2007**;35:3869-78
3. Zhou T, Akopiants K, Mohapatra S, Lin PS, Valerie K, Ramsden DA*, et al.* Tyrosyl-DNA phosphodiesterase and the repair of 3'-phosphoglycolate-terminated DNA double-strand breaks. DNA Repair (Amst) **2009**;8:901-11
4. Golding SE, Rosenberg E, Khalil A, McEwen A, Holmes M, Neill S*, et al.* Double strand break repair by homologous recombination is regulated by cell cycle-independent signaling via ATM in human glioma cells. J Biol Chem **2004**;279:15402-10
5. Golding SE, Rosenberg E, Neill S, Dent P, Povirk LF, Valerie K. Extracellular signal-related kinase positively regulates ataxia telangiectasia mutated, homologous recombination repair, and the DNA damage response. Cancer Res **2007**;67:1046-53
6. Mateos-Gomez PA, Gong F, Nair N, Miller KM, Lazzerini-Denchi E, Sfeir A. Mammalian polymerase theta promotes alternative NHEJ and suppresses recombination. Nature **2015**;518:254-7
7. Wyatt DW, Feng W, Conlin MP, Yousefzadeh MJ, Roberts SA, Mieczkowski P*, et al.* Essential Roles for Polymerase theta-Mediated End Joining in the Repair of Chromosome Breaks. Mol Cell **2016**;63:662-73
8. Sanjana NE, Shalem O, Zhang F. Improved vectors and genome-wide libraries for CRISPR screening. Nat Methods **2014**;11:783-4
9. Stringer BW, Day BW, D'Souza RCJ, Jamieson PR, Ensbey KS, Bruce ZC*, et al.* A reference collection of patient-derived cell line and xenograft models of proneural, classical and mesenchymal glioblastoma. Sci Rep **2019**;9:4902
10. Koebley SR, Reed J. Can a new microscopy platform help to improve clinical outcomes? Biotechniques **2018**;65:250-1
11. Mikheikin A, Olsen A, Leslie K, Russell-Pavier F, Yacoot A, Picco L*, et al.* DNA nanomapping using CRISPR-Cas9 as a programmable nanoparticle. Nat Commun **2017**;8:1665
12. Mikheikin A, Olsen A, Picco L, Payton O, Mishra B, Gimzewski JK*, et al.* High-Speed Atomic Force Microscopy Revealing Contamination in DNA Purification Systems. Anal Chem **2016**;88:2527-32
13. Binnig G, Quate CF, Gerber C. Atomic force microscope. Phys Rev Lett **1986**;56:930-3
14. Picco LM, Dunton PG, Ulcinas A, Engledew DJ, Hoshi O, Ushiki T*, et al.* High-speed AFM of human chromosomes in liquid. Nanotechnology **2008**;19:384018
15. Canman CE, Lim DS, Cimprich KA, Taya Y, Tamai K, Sakaguchi K*, et al.* Activation of the ATM kinase by ionizing radiation and phosphorylation of p53. Science **1998**;281:1677-9

16. Campbell KS, Auger KR, Hemmings BA, Roberts TM, Pallas DC. Identification of regions in polyomavirus middle T and small t antigens important for association with protein phosphatase 2A. J Virol **1995**;69:3721-8
17. Einav Y, Shistik E, Shenfeld M, Simons AH, Melton DW, Canaani D. Replication and episomal maintenance of Epstein-Barr virus-based vectors in mouse embryonal fibroblasts enable synthetic lethality screens. Mol Cancer Ther **2003**;2:1121-8
